# Endogenous viral elements reveal associations between a non-retroviral RNA virus and symbiotic dinoflagellate genomes

**DOI:** 10.1038/s42003-023-04917-9

**Published:** 2023-06-01

**Authors:** Alex J. Veglia, Kalia S. I. Bistolas, Christian R. Voolstra, Benjamin C. C. Hume, Hans-Joachim Ruscheweyh, Serge Planes, Denis Allemand, Emilie Boissin, Patrick Wincker, Julie Poulain, Clémentine Moulin, Guillaume Bourdin, Guillaume Iwankow, Sarah Romac, Sylvain Agostini, Bernard Banaigs, Emmanuel Boss, Chris Bowler, Colomban de Vargas, Eric Douville, Michel Flores, Didier Forcioli, Paola Furla, Pierre E. Galand, Eric Gilson, Fabien Lombard, Stéphane Pesant, Stéphanie Reynaud, Shinichi Sunagawa, Olivier P. Thomas, Romain Troublé, Didier Zoccola, Adrienne M. S. Correa, Rebecca L. Vega Thurber

**Affiliations:** 1 BioSciences Department, Rice University, Houston, TX USA; 2 Microbiology Department, Oregon State University, Corvallis, OR USA; 3 Department of Biology, University of Konstanz, Konstanz, Germany; 4 Department of Biology, Institute of Microbiology and Swiss Institute of Bioinformatics, Vladimir-Prelog-Weg 4, ETH Zürich, CH-8093 Zürich, Switzerland; 5 PSL Research University: EPHE-UPVD-CNRS, USR 3278 CRIOBE, Laboratoire d’Excellence CORAIL, Université de Perpignan, 52 Avenue Paul Alduy, 66860 Perpignan, Cedex France; 6 Centre Scientifique de Monaco, 8 Quai Antoine Ier, Monaco, MC-98000 Principality of Monaco; 7 Génomique Métabolique, Genoscope, Institut François Jacob, CEA, CNRS, Univ Evry, Université Paris-Saclay, Evry, France; 8 Research Federation for the study of Global Ocean Systems Ecology and Evolution, FR2022/ Tara Oceans-GOSEE, 3 rue Michel-Ange, 75016, Paris, France; 9 Fondation Tara Océan, Base Tara, 8 rue de Prague, 75012 Paris, France; 10 School of Marine Sciences, University of Maine, Orono, ME USA; 11 Sorbonne Université, CNRS, Station Biologique de Roscoff, AD2M, UMR 7144 ECOMAP, Roscoff France; 12 Shimoda Marine Research Center, University of Tsukuba, 5-10-1 Shimoda, Shizuoka Japan; 13 Institut de Biologie de l’Ecole Normale Supérieure (IBENS), Ecole normale supérieure, CNRS, INSERM, Université PSL, 75005 Paris, France; 14 Laboratoire des Sciences du Climat et de l’Environnement, LSCE/IPSL, CEA-CNRS-UVSQ, Université Paris-Saclay, F-91191 Gif-sur-Yvette, France; 15 Weizmann Institute of Science, Department of Earth and Planetary Sciences, 76100 Rehovot, Israel; 16 Université Côte d’Azur, CNRS, INSERM, IRCAN, Medical School, Nice, France; 17 Laboratoire International Associé Université Côte d’Azur-Centre Scientifique de Monaco, LIA ROPSE, Monaco, France; 18 Sorbonne Université, CNRS, Laboratoire d’Ecogéochimie des Environnements Benthiques (LECOB), Observatoire Océanologique de Banyuls, 66650 Banyuls sur mer, France; 19 Department of Medical Genetics, CHU of Nice, Nice, France; 20 Sorbonne Université, Institut de la Mer de Villefranche sur mer, Laboratoire d’Océanographie de Villefranche, F-06230 Villefranche-sur-Mer, France; 21 European Molecular Biology Laboratory, European Bioinformatics Institute, Wellcome Genome Campus, Hinxton, Cambridge, CB10 1SD UK; 22 School of Biological and Chemical Sciences, Ryan Institute, University of Galway, University Road H91 TK33, Galway, Ireland

**Keywords:** Water microbiology, Microbial ecology, Virology

## Abstract

Endogenous viral elements (EVEs) offer insight into the evolutionary histories and hosts of contemporary viruses. This study leveraged DNA metagenomics and genomics to detect and infer the host of a non-retroviral dinoflagellate-infecting +ssRNA virus (dinoRNAV) common in coral reefs. As part of the Tara Pacific Expedition, this study surveyed 269 newly sequenced cnidarians and their resident symbiotic dinoflagellates (Symbiodiniaceae), associated metabarcodes, and publicly available metagenomes, revealing 178 dinoRNAV EVEs, predominantly among hydrocoral-dinoflagellate metagenomes. Putative associations between Symbiodiniaceae and dinoRNAV EVEs were corroborated by the characterization of dinoRNAV-like sequences in 17 of 18 scaffold-scale and one chromosome-scale dinoflagellate genome assembly, flanked by characteristically cellular sequences and in proximity to retroelements, suggesting potential mechanisms of integration. EVEs were not detected in dinoflagellate-free (aposymbiotic) cnidarian genome assemblies, including stony corals, hydrocorals, jellyfish, or seawater. The pervasive nature of dinoRNAV EVEs within dinoflagellate genomes (especially *Symbiodinium*), as well as their inconsistent within-genome distribution and fragmented nature, suggest ancestral or recurrent integration of this virus with variable conservation. Broadly, these findings illustrate how +ssRNA viruses may obscure their genomes as members of nested symbioses, with implications for host evolution, exaptation, and immunity in the context of reef health and disease.

## Abstract (web summary)

A study part of the Tara Pacific Expedition that surveyed newly sequenced and publicly available metagenomes and genomes revealed pervasive non-retroviral dinoflagellate-infecting endogenous +ssRNA viral elements within coral symbionts.

## Introduction

Endogenous viral elements, or “EVEs,” arise when whole or fragmented viral genomes are incorporated into host cell germlines. Once integrated, EVEs may propagate across successive host generations, potentially becoming fixed in a population through natural selection or drift^1,2^. Therefore, the presence and content of EVEs can provide clues into the evolutionary relationships among host species and shed light on ancient and modern virus-host interactions^3^. To date, most EVEs described in metazoan and plant genomes are retroviral, as this viral group must integrate their genome (as a provirus) into the genome of the host to replicate. Retroviruses thus possess and encode all of the molecular machinery (e.g. reverse transcriptases, integrases) required to integrate autonomously^4^. Remarkably, however, sequences from viruses that do not encode reverse transcriptases or exploit integration as a component of an obligate replication strategy—even viruses with no DNA stage—have also recently been detected as EVEs in diverse eukaryotic genomes^5–11^. These non-retroviral RNA EVEs have been reported in hosts ranging from unicellular algae to chiropteran (bat) genomes^12–18^. Though the mechanisms behind non-retroviral integration continue to be explored, viral sequences may be introduced via nonhomologous recombination and repair, through interactions with host-provisioned integrases and reverse transcriptases supplied on mobile elements (e.g. retroelements), or by utilizing co-infecting viruses^6,7^.

Endogenization of any viral sequence (including non-retroviral EVEs) may have positive, neutral or negative effects on a host^19–21^. While many EVEs are functionally defective or deleterious and ultimately removed from a population via purifying selection, retained EVEs may remodel the genomic architecture of their hosts or introduce sources of genetic innovation later co-opted for host function (i.e. exaptation^22,23^). Such ‘domesticated’ EVEs can be co-opted by hosts and utilized as regulatory elements, transcription factors, or functional proteins with purposes ranging from organism development to synaptic plasticity in the mammalian brain^24–28^. In particular, non-retroviral EVEs potentially serve as antiviral prototypes that help hosts combat infection by exogenous viruses currently circulating in the population^14,29–31^. Mechanisms underpinning EVE-derived immunity can include cell receptor interference, nucleic acid sequence recognition (e.g., RNAi), or even replication sabotage through production of faulty virus proteins from EVEs^32^. If expressed, EVEs may have a significant influence on the health, physiology and/or behavior of their hosts in natural and experimental systems^31,33,34^.

Investigating the distribution, sequence identity, and function of EVEs can yield insight into virus-host interactions across generations. EVEs catalogue a subset of the viruses that a host lineage has encountered and can link homologous extant viruses to contemporary hosts or known disease states^31^. Because integrated elements may accrue mutations at a slower rate than exogenous viral genomes^6,35^, EVEs can fill gaps in virus-host networks and act as synapomorphies, indicating the minimum time that a virus may have interacted with a host. As ‘genomic fossils’, EVEs have helped paleovirologists date the minimum origin of *Circoviridae*^36^, *Hepadnaviridae*^37^, *Bornaviridae*^38^, *Flaviviridae*^39^, *Lentiviridae*^40,41^, and *Spumaviridae*^42^ infections within metazoans^2,24,35,43,44^ (reviewed by Barreat and Katzourakis in 2022^45^).

Coral holobionts – the cnidarian animal and its resident microbial assemblage, including dinoflagellates in the family Symbiodiniaceae, bacteria, archaea, fungi, and viruses – are an ecologically and economically valuable, multipartite non-model system^46,47^. Symbiodiniaceae are key obligate nutritional symbionts of corals and support their hosts in the construction of reef frameworks^48^. However, environmental stress can break down coral-Symbiodiniaceae partnerships, resulting in bleaching – the mass loss of Symbiodiniaceae cells^49^. Some bleaching signs (paling of a coral colony) are hypothesized to also result from viral lysis of Symbiodiniaceae^21,50–54^, but direct evidence supporting this hypothesis remains limited. Overall, the role of viruses in coral colony health and disease requires further examination.

Non-retroviral +ssRNA dinoRNAV sequences were first reported in stony corals based on five metatranscriptomic sequences and corroborated by Symbiodiniaceae EST libraries^55^. Subsequent studies indicated that similar +ssRNA viruses are commonly detected in coral RNA viromes and metatranscriptomes, as well as via targeted amplicon assays^54,56–58^. These viruses exhibit synteny and significant homology to *Heterocapsa circularisquama* RNA virus (HcRNAV^57^), the sole recognized representative of the genus *Dinornavirus* and a known pathogen of free-living dinoflagellates^59^. Both HcRNAV and dinoRNAV sequences detected in coral holobiont tissues contain two ORFs – a Major Capsid Protein (*MCP)* and RNA dependent RNA polymerase (*RdRp*). Furthermore, icosahedral virus-like particle (VLP) arrays resembling HcRNAV (but with 40% smaller individual particle diameters) have been imaged in the Symbiodiniaceae-dense coral gastrodermis tissue and in Symbiodiniaceae themselves^60^. Levin et al. (2017)^57^ assembled the 5.2 kb genome of a putative dinoRNAV from a poly(A)-selected metatranscriptome generated from cultured *Symbiodinium*. The assembly contained a 5’ dinoflagellate spliced leader (“dinoSL”^61^) — a component of >95% of Symbiodiniaceae mRNAs, speculated to illustrate molecular mimicry — and exhibited >1000-fold higher expression in a thermosensitive *Cladocopium C1* population relative to a thermotolerant population of this Symbiodiniaceae strain at ambient temperatures (27 °C^48,57^). Together, the findings from these studies suggest that Symbiodiniaceae are target hosts of reef-associated dinoRNAVs.

This study (1) systematically searched for putative endogenized dinoRNAVs in metagenomes from in situ (symbiotic) coral colonies and seawater, as well as in available genomes of Symbiodiniaceae and aposymbiotic (symbiont-free) cnidarians, (2) investigated the evolutionary relationship of putative dinoRNAV EVEs to exogenous reef-associated dinoRNAV sequences, and (3) made preliminary inferences regarding the distribution and possible function of these dinoRNAV EVEs based on their detection, prevalence, and genomic context.

## Results and discussion

### Evidence of endogenized dinoRNAVs in coral holobiont metagenomes

**Fig. 1:**
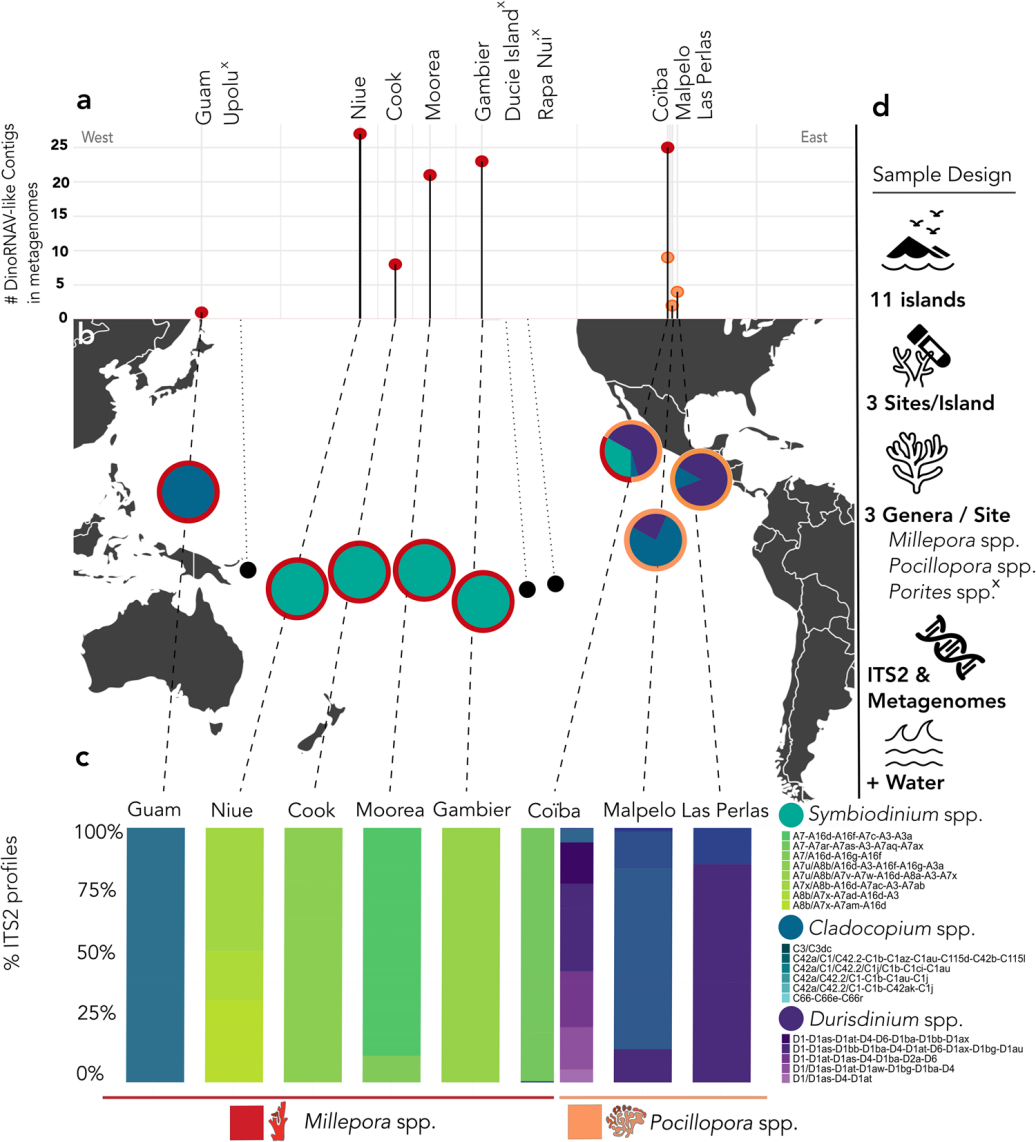
Islands and species (cnidarian and dinoflagellate) correlating with dinoRNAV EVE-like sequence detection among Tara Pacific metagenomes. **a** Count of scaffolds with putative endogenized dinoRNAV-like sequences among Tara Pacific metagenomes, grouped by island and spaced longitudinally by location sampled. **b** Sampling sites of Tara Pacific metagenomes explored for endogenized dinoRNAV-like sequences in this study. Internal circles indicate dominant Symbiodiniaceae genera based on ITS2 type profiles, outer ring denotes coral host(s) sampled at each island. **c** Symbiodiniaceae ITS2 type profile metabarcoding as delineated via Symportal^119^ within island and host. **d** Sample design of Tara Pacific libraries queried for dinoRNAV EVEs. [x] and black circles on map indicate island locations or species where no dinoRNAV-like sequences were detected. Icons derived from the Noun Project.

**Fig. 2:**
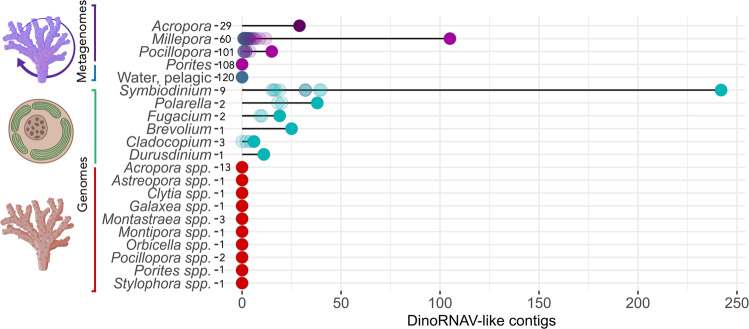
Quantity of putative endogenized dinoRNAV per metagenome or genome. Total quantity of putative endogenized dinoRNAV EVEs identified, broadly organized by sample source (metagenome or genome), and number of libraries or assemblies queried (after source name, Supplementary Data 6). Opaque circles denote the sum total of dinoRNAV EVE-like sequences identified from each source, while transparent circles denote individual counts of putative dinoRNAV EVEs per library. Icons created with BioRender.

Putative dinoRNAV EVEs were detected in metagenomes generated from 42 cnidarian holobionts out of 269 sampled across the South Pacific Ocean (Supplementary Data 1). The majority of endogenized dinoRNAVs were identified in hydrocoral metagenomes (*Millepora* spp.; 70.5%, *n* = 105) which predominantly harbored *Symbiodinium* dinoflagellates but EVE-like sequences were also observed in scleractinian coral metagenomes (*Pocillopora* spp.; 29.5%, *n* = 15.) which predominantly harbored *Cladocopium* and *Durusdinium* dinoflagellates (Fig. 1a, c). No dinoRNAV-like sequences were detected among *Porites* spp. metagenomes (Figs. 1,  2). Hydrocoral metagenomes were sequenced at equivalent depths as scleractinian corals and had comparable levels of annotation (Supplementary Fig. 1, Supplementary Data 2); thus, higher dinoRNAV EVE prevalence in hydrocoral libraries was likely not a result of methodological bias. Of the 11 evaluated South Pacific islands, dinoRNAV EVEs were identified in samples from eight (Guam, Gambier, Moorea, Cook, Niue, Malpelo, Coïba, and Las Perlas), spanning 18 unique sites (Fig. 1b, d). Among *Pocillopora* spp. metagenomes, putative dinoRNAV EVEs were only identified on the Central American coast (CAMR, Coastal Pacific Longhurst Province) and were absent in Melanesia, Micronesia, and Polynesia; at these latter sites, dinoRNAVs were largely found in *Millepora* hydrocoral metagenomes. Importantly, endogenized dinoRNAV open reading frames (ORFs) appeared to be immediately adjacent to ORFs identified as dinoflagellate (typically Symbiodiniaceae) genes—they were not proximal to coral genes or those of other cellular organisms abundant in these metagenomes (Supplementary Data 3).

We examined the Symbiodiniaceae ITS2 profiles associated with each metagenome and found that putative dinoRNAV EVEs were primarily associated with *Symbiodinium*, *Cladocopium*, and *Durusdinium*, which exhibited variation on both host and regional scales (Fig. 1c, Supplementary Data 4). DinoRNAV EVEs were more common in *Symbiodinium*-dominated cnidarians (*F*_*2,1044*_ = *25.8*, *p* < 0.0001, nested ANOVA; Supplementary Fig. 2, Supplementary Data 5) relative to cnidarians hosting other Symbiodiniaceae genera, regardless of host. This suggested that dinoRNAV integration may be particularly recurrent or conserved within the genus *Symbiodinium* (Fig. 1).

To determine if these putative viral integrations were specific to cnidarian holobiont metagenomes and ensure that they were not artifacts of shared sample processing and sequencing procedures of the Tara Pacific pipeline, we also analyzed seawater metagenomes and publicly available metagenomes from the stony coral-dinoflagellate holobiont, *Acropora* spp. (Supplementary Data 1B,^62–69^). Examination of 120 Tara Oceans pelagic seawater metagenomes^70^ yielded no sequences sharing homology to dinoRNAVs. The concentration of Symbiodiniaceae cells within cnidarian tissues is considerably higher than that of the surrounding seawater^71–74^. On average, only 1.46 ± 0.08% of assembled contigs in seawater metagenomes were annotated as Symbiodiniaceae. Thus, lack of detection of dinoRNAV-like sequences from seawater metagenomes is likely due to reduced genomic signal of Symbiodiniaceae in the water column, rather than a lack of EVEs associated with Symbiodiniaceae lineages in seawater. However, it also must be noted that these Tara Oceans seawater metagenomes were not collected concurrently with coral samples^75^. Analysis of the 30 non-Tara *Acropora* holobiont metagenomes identified 29 more putative dinoRNAV EVEs (Fig. 2; Supplementary Data 6). These dinoRNAV EVEs were again neighboring dinoflagellate ORFs. While the Caribbean *Acropora* metagenomes analyzed contained too few reads to resolve the dominant Symbiodiniaceae present, earlier studies of the same coral colonies identified *Symbiodinium* spp. as the primary symbiont present^76^.

The identification of endogenized dinoRNAV-like sequences in cnidarian holobiont metagenomes, combined with the proximity of dinoRNAV-like ORFs to dinoflagellate-like sequences across metagenomes harboring diverse dinoflagellate consortia, collectively indicate that dinoRNAV EVEs are widespread among Symbiodiniaceae genera (Fig. 2 cyan dots).

### Endogenized DinoRNAVs detected in Symbiodiniaceae genomes

To further test the hypothesis that dinoRNAVs on reefs infect dinoflagellate symbionts and not cnidarians, we examined 18 scaffold-scale genome assemblies representing the dinoflagellate families Symbiodiniaceae and Suessiaceae as well as 25 cnidarian genomes spanning 10 genera (Supplementary Data 1B^62–69^; Fig. 2; Table 1). Alignments revealed no evidence of endogenized dinoRNAVs in any of the 151,782 aposymbiotic (dinoflagellate-free) cnidarian scaffolds. In contrast, the same approach uncovered 351 (of 593,433) dinoflagellate scaffolds with evidence of endogenized dinoRNAVs (Fig. 2; Table 1). The identified 351 dinoRNAV EVE-containing scaffolds were observed across 17 of the 18 dinoflagellate genome assemblies (Table 1). DinoRNAV EVEs were also observed in two assemblies from the free-living dinoflagellate genus, *Polarella* (family Suessiaceae), which is closely related to the family Symbiodiniaceae, and served as an outgroup in this study^77,78^. Interestingly, assemblies belonging to *Symbiodinium*, the most ancestral Symbiodiniaceae genus^48^, contained a higher number of scaffolds with putative dinoRNAV EVEs (x̄=28.11, stdev=10.7) relative to assemblies of other Symbiodiniaceae genera (x̄=8.71, stdev=11; Fig. 2 cyan dots; Table 1). This result may clarify why observations of dinoRNAV-like ORFs were more common in metagenomes dominated by *Symbiodinium* (Fig. 1c). The dinoflagellate genome assembly with no detected dinoRNAV EVEs belonged to a relatively incomplete assembly of *Cladocopium* C15, which had the second lowest N50 and lowest BUSCO completeness score of all genomes examined (completeness 11.6%, relative to the average 24.54%; Table 1, Supplementary Data 7). The lower coverage/completeness of the *Cladocopium* C15 assembly indicates a reduced window into this genome. It is therefore possible that when a more complete assembly is generated, dinoRNAV EVE-like sequences will be detectable from this dinoflagellate. However, a linear model suggested that there was no relationship between dinoRNAV EVE detection and assembly statistics (i.e. query length, N50, or completeness; see Supplementary Data 8 for linear model output). Instead, dinoflagellate genus was the strongest predictor of dinoRNAV detection in a genome (LM results: Genus *F* = 5.74, *p* = 0.012) and dinoRNAV detections were significantly higher in *Symbiodinium* than *Cladocopium* genomes (pairwise estimated difference = −27.77 ± 5.91, *p* = 0.01; Supplementary Data 9). Furthermore, since we were unable to detect dinoRNAV EVEs in *Porites* metagenomes—a coral species primarily harboring *Cladocopium* C15 symbionts – we hypothesize that dinoRNAV endogenization was either less common in this lineage of Symbiodiniaceae or integrations have been lost over evolutionary time^79,80^.

**Table 1:** DinoRNAV EVE-like open reading frames from representative Symbiodiniaceae and Suessiaceae dinoflagellate scaffold-level genome assemblies.

	Dinoflagellate Species (strain)	Total # Scaffolds	Host	Location	BUSCO score	dinoRNAV EVE ORFs on scaffolds		
						*RdRp*	*MCP*	Both
Symbiodiniaceae	*Symbiodinium linucheae* (CCMP2456)^83^	37,772	*Plexaura homamalla*	Bermuda	21.8%	39	0	1
	*Symbiodinium microadriaticum* (04-503SCI.03)^83^	57,558	*Orbicella faveolata*	Florida, USA	41.6%	30	1	3
	*Symbiodinium microadriaticum* (CassKB8)^83^	67,937	*Cassiopea sp*.	Hawaii, USA	73.3%	29	1	3
	*Symbiodinium microadriaticum* (CCMP2467)^113^	9688	*Stylophora pistillata*	Red Sea	15.6%	29	1	3
	*Symbiodinium natans* (CCMP2548)^83^**	2855	N/A (Isolated from seawater)	Hawaii, USA	15.5%	14	1	3
	*Symbiodinium necroappetens* (CCMP2469)^83^*	104,583	*Condylactis gigantea*	Jamaica	22.8%	37	4	2
	*Symbiodinium pilosum* (CCMP2461)^83^**	48,302	*Zoanthus sociatus*	Jamaica	19.8%	15	0	0
	*Symbiodinium* sp. A5 (formerly *S. tridacnidorum*)^83^	6245	*Heliofungia actiniformis*	Australia	21.1%	17	1	0
	*Symbiodinium tridacnidorum* (sh18 A3 Y106)^114^	16,176	*Tridacna crocea*	Japan	19.8%	20	1	0
	*Brevolium minutum* (Mf1.05b)^115^	21,899	*Orbicella faveolata*	Florida, USA	14.2%	21	3	1
	*Cladocopium* C15^116^	34,589	*Porites lutea*	Australia	11.6%	0	0	0
	*Cladocopium* sp. *C1acro* (formerly *C. goreaui*)^89^	41,289	*Acropora tenuis*	Australia	27.7%	4	0	0
	*Cladocopium sp* C92 (Y103)^114^	6686	*Fragum* sp.	Japan	19.5%	2	0	0
	Durusdinium trenchii^117^	19,593	*Favia speciosa*	Japan	28.7%	10	1	0
	*Fugacium kawagutti* (CS156 CCMP2468)^89^	16,959	N/A (Free-living)	Hawaii, USA	8.3%	8	1	0
	*Fugacium kawagutti* (CCMP2468)^118^	30,040	N/A (Free-living)	Hawaii, USA	17.9%	9	1	0
*n *= 314 scaffolds with DinoRNAV EVE-like sequences								
Suessiaceae	*Polarella glacialis* (CCMP1383)^78^ **	33,494	N/A (Free-living, isolated from seawater)	Antarctica	20.8%	20	0	0
	*Polarella glacialis* (CCMP2088)^78^ **	37,768	N/A (Free-living, isolated from seawater)	Arctic	21.8%	18	0	0
*n* = 38 total scaffolds with DinoRNAV EVE-like sequences								

Total counts of dinoflagellate scaffolds in genomes queried with individual endogenized dinoRNAV ORFs (*RdRp*, *MCP*) or both nearby each other, potentially indicating full viral genome integration. Table supplies associated dinoflagellate host species and location of isolation. *RdRp* = RNA-dependent RNA polymerase; *MCP* = major capsid protein. Assembly coverage and completeness are measured via BUSCO score (% completeness, or %C)^102^. * Indicates species with documented opportunistic life history; ** Indicates species with documented free-living life history per principal species description. Gonzalez-Pech et al.^83^, Aranda et al.^113^, Shoguchi et al.^114^, Shoguchi et al.^115^, Robbins et al.^116^, Liu et al.^89^, Shoguchi et al.^117^, Lin et al.^118^, Stephens et al.^78^. Further genome citations (including accession numbers) and BUSCO completion metrics can be found in Supplementary Data 7 and in superscript.

### Incomplete ORFs and possible duplications indicate endogenization of DinoRNAVs

The repeated observation of putative dinoRNAV EVEs in dinoflagellate scaffolds and contigs from metagenomes and genomes suggests these sequences are either (1) conserved sequence artifacts of Symbiodiniaceae-dinoRNAV interactions, and/or (2) evidence of highly prevalent dinoflagellate viruses, commonly integrated and propagated via their single-celled hosts. If the observed dinoRNAV-like sequences represent active infections capable of generating virions during egress, we would, at minimum, expect essential ORFs associated with replication (RNA-dependent RNA polymerase, *RdRp*) and virion structure (Major Capsid Protein, *MCP*) to be endogenized on the same scaffold. We would additionally expect to observe overall conservation of ORF length/composition (with a lack of internal stop codons or substantial deletions) when aligning the dinoRNAV-like sequences detected here with known exogenous dinoRNAV sequences.

**Fig. 3:**
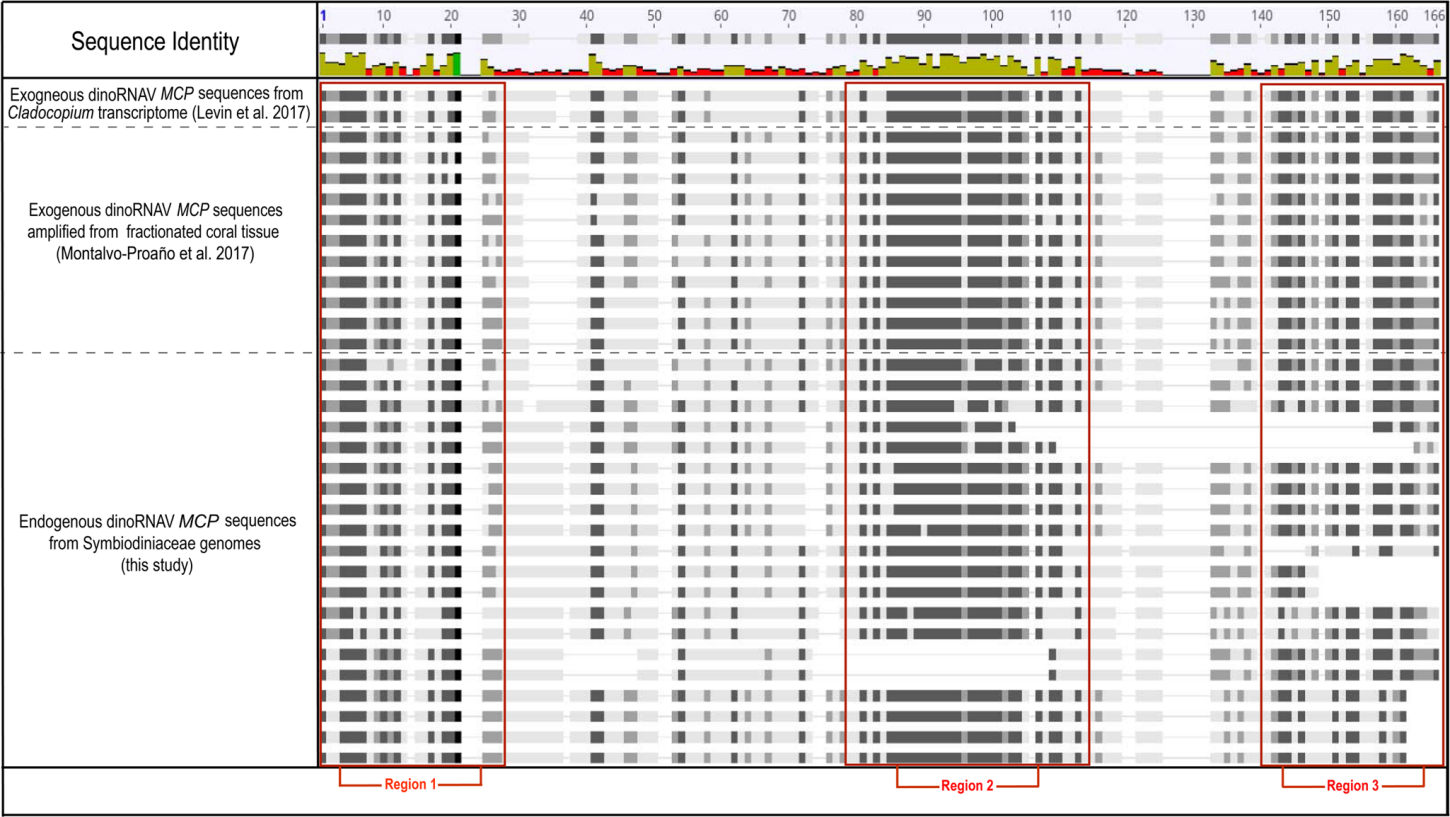
Amino acid alignment of putative endogenous and exogenous dinoRNAV-like +ssRNA virus Major Capsid Protein (MCP) sequences against transcriptome reference. Putatively endogenous dinoRNAV Major Capsid Protein (MCP) amino acid sequences were aligned against exogenous references, including: (1) Symbiodiniaceae +ssRNA virus MCP ORFs recovered from a *Cladocopium sp*. transcriptome (Levin et al, 2017), and (2) dinoRNAV MCP amplicons from fractionated coral tissue (Montalvo-Proaño et al. 2017). Conserved regions were observed between exogenous and putatively endogenous viral sequences (labeled Regions 1–3).

However, both DIAMOND and gene prediction analyses generally depicted dinoRNAV-like ORFs in isolation on separate scaffolds. While 28 *MCP* and 73 *RdRp* dinoRNAV ORFs were annotated, both ORFs were present on a Symbiodiniaceae scaffold – potentially representing whole dinoRNAV genome integrations – in only 14 instances. Thirteen of these 14 were from *Symbiodinium* genomes, whereas one scaffold was from *Breviolum minutum*, a member of the second most ancestral dinoflagellate genus (Table 1)^48^. To assess the conservation of putative dinoRNAV EVE sequence length/composition, we aligned the genomic and single ORF EVEs to reference exogenous dinoRNAV sequences. The reference genome for reef-associated dinoRNAVs is ~5 Kbp long and contains a 1,071 bp noncoding region between ORFs, with a 124-nucleotide internal ribosomal binding site^57^. In this study, for 13 of the scaffolds in which dinoRNAV ORFs were detected, the putative noncoding region between the *MCP* and *RdRp* EVEs ranged from ~200-800 bp (except for a scaffold belonging to *S. linucheae* CCMP2456, which contained a ~ 79 kbp noncoding region, and was excluded in further alignments). No internal ribosomal binding sites were detected within the putative dinoRNAV EVEs identified in dinoflagellate genomes. A nucleotide-based alignment to Levin et al.’s (2017)^57^ reference dinoRNAV genome indicated that the putative dinoRNAV EVEs presented here contained substantial insertions and/or deletions (Supplementary Fig. 3). Translated exogenous dinoRNAV MCP ORFs are reported to be ~358 aa in length^57^; Fig. 3 top sequences), but dinoRNAV-like MCP sequences recovered in this study ranged from 116-605aa in length. Furthermore, comparisons of these endogenous MCPs to exogenous reference sequences revealed internal stop codons and overall low similarity (Fig. 3). Amino acid-based alignment of endogenous dinoRNAV MCPs to metatranscriptome- and amplicon-generated exogenous reference sequences^57,58^ revealed indels and regions of low similarity between three conserved regions across both endogenous and exogenous MCP sequences (red boxes in Fig. 3).

Interestingly, multiple whole dinoRNAV integrations were sometimes observed in a single dinoflagellate genome. For example, genome assemblies of four different *S. microadriacticum* strains contained two or three whole dinoRNAV EVEs each (Table 1; Fig. 2). Pairwise alignments measuring shared nucleotide identity of whole dinoRNAV EVEs across Symbiodiniaceae scaffolds revealed that the *S. microadriaticum* genomes and the *S. necroappetens* genome share two whole genome dinoRNAV EVEs (provisionally dinoRNAV-A and dinoRNAV-B; Supplementary Fig. 3; Clustal-Omega)^81^. *S. microadriaticum* dinoRNAV-B was identical in all strains and shared 97% identity with the *S. necroappetens* dinoRNAV-B, yet proximal genes varied (Supplementary Data 10, 11). Importantly, the inconsistent composition and fragmented nature of both the genomic and single ORF dinoRNAV EVEs reported here supports the hypothesis that these sequences are not capable of generating replicative virions and are best interpreted as multiple integrations of dinoRNAVs into a host genome.

### A Potential Mechanism for dinoRNAV Endogenization: Host-Provisioned Retroelements

**Fig. 4:**
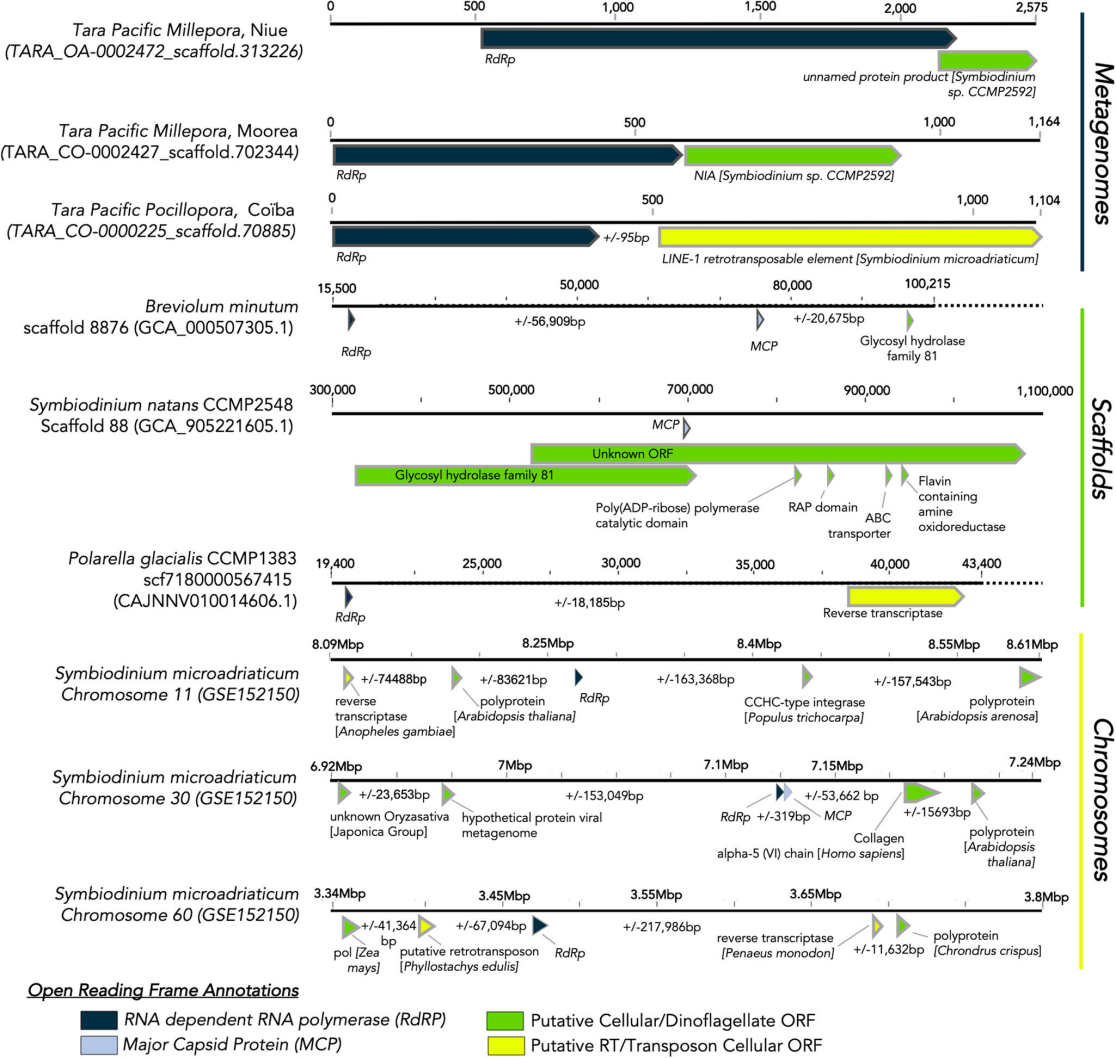
Representative scaffolds and chromosome fragments containing putative dinoRNAV EVEs. Scaffolds annotation: *MCP* ORFs are indicated by light blue, *RdRp* indicated by navy blue ORFs (with complete description in Supplementary Data 10). Open reading frame (ORF) color broadly indicates cellular versus putative +ssRNA viral homology; yellow and some green (e.g., integrases, polyproteins) ORFs may be exploited mechanisms for viral integration. (+/−) base pair values represent sequence lengths between ORFs.

To assess if general genomic “neighborhoods” are conserved across dinoRNAV integrations (e.g. site location and synteny) and to better understand the genes proximal to EVEs on Symbiodiniaceae genomes, a chromosome-scale *Symbiodinium microadriaticum* genome assembly was evaluated (Fig. 4). The highest quality dinoflagellate genome assembly currently available revealed dinoRNAV-like ORFs on 18 of 94 chromosomes, with at least one *RdRp* on each, and some with multiple (two with *n* = 2 *RdRps*, three with *n* = 3 *RdRps*). On three of the chromosomes (# 30, 35, and 74), there were predicted ORFs annotated as dinoRNAV *MCPs* in close proximity to a *RdRp* ORF (separated by noncoding regions 319-656nt), indicative of a potential full-length dinoRNAV genome integration. These results corroborate detections of multiple genomic dinoRNAV EVEs in scaffold-scale assemblies of *Symbiodinium microadriaticum* genomes (Supplementary Fig. 3). The higher-resolution *S. microadriaticum* chromosome-level assembly facilitated the identification of an additional dinoRNAV genomic EVE (*n* = 4 for chromosome-level vs. *n* = 3 for scaffold-level, Supplementary Fig. 3), two of which were identified on Chromosome 74 and were separated by 2501 nucleotides. Of note, Nand et al. (2021)^82^ reported a decreasing abundance and expression of genes towards the center of chromosomes (past ~2Mpb of a telomere), where there was an increase in repetitive elements; this is where 26 of 29 putative dinoRNAV EVEs were identified in the chromosome-level assembly. Furthermore, ORFs neighboring integrations often varied widely, both in proximity and predicted function, from collagen and RNA binding protein to reverse transcriptase and non-LTR retrotransposable elements. These ORFs potentially contributed to the endogenization of dinoRNAV via mechanisms such as retrotransposition (Fig. 4, Supplementary Data 10).

Retroposition through host-provisioned retroelements is one proposed mechanism of non-retroviral RNA virus integration into eukaryotic genomes^6,7^. An indicator of this form of integration is the nearby presence of a relict dinoflagellate spliced leader (“dinoSL”), a 22nt sequence located at the 5’ end of mRNAs^83–86^. Such a sequence flanks the *RdRp* gene on some extant dinoRNAVs^57^. We detected dinoSLs within 500 bp of 23.1% (six of 26) endogenized *RdRp* ORFs on *S. micoradriaticum* chromosomes, providing support for retroposition of these viral elements into Symbiodiniaceae genomes (Supplementary Data 11, 12). DinoRNAV gene integration may be facilitated by any of three major orders of retroelements associated with Symbiodiniaceae, including long terminal repeat (LTR) retrotransposons, short interspersed nuclear elements (SINEs), and long interspersed nuclear elements (LINEs^83,87,88^). Evidence suggests that these LINEs are common and non-active remnants of an ancient proliferation of LINEs that preceded the diversification of Suessiales^78,83,89^. *Symbiodinium* contains more LINEs relative to other Symbiodiniaceae genera, comprising 74.10-171.31 Mbp of *Symbiodinium* genomes, relative to an average of 7.48 Mbp of the genomes of in other genera, indicating the loss of these retroelements across speciation events^82,83^. The loss of LINEs in more recently derived Symbiodiniaceae genera coincides with a decrease in dinoRNAV EVE detection in these genomes (Table 1). Conversely, the genomes of *Polarella*, the psychrophilic and free-living outgroup from which Symbiodiniaceae diversified ~160 million years ago, are LINE-rich and generally have comparable numbers of dinoRNAV EVEs to *Symbiodinium* (Table 1^48,77,78,83^). Together, this suggests that LINE activity during speciation may have facilitated dinoRNAV integration and the resulting EVEs may constitute dinornavirus “fossils.” This may explain their degree of sequence fragmentation and relatively low sequence similarity to modern extant dinoRNAVs (Fig. 3).

LINE-mediated retroposition is further supported by the observation of a LINE reverse transcriptase homolog ~17 kbp upstream of a *RdRp* EVE with a relict dinoSL on chromosome 45 (Supplementary Data 12) and a LINE retroelement 95 bp downstream of an EVE recovered from a *Pocillopora* metagenome (Fig. 4). Additionally, ~40% of annotated ORFs (35 of 88 annotated proteins) proximal to dinoRNAV ORFs on *S. microadriaticum* chromosomes were similar to non-LTR elements seen in other eukaryotic genomes sometimes <300 bp 5’ upstream (Supplementary Data 12, Supplementary Fig. 4). Collectively, these findings implicate host provisioned retroelements, such as LINEs, as facilitators of dinoRNAV gene integration.

### DinoRNAV EVEs show homology to extant exogenous viruses

**Fig. 5:**
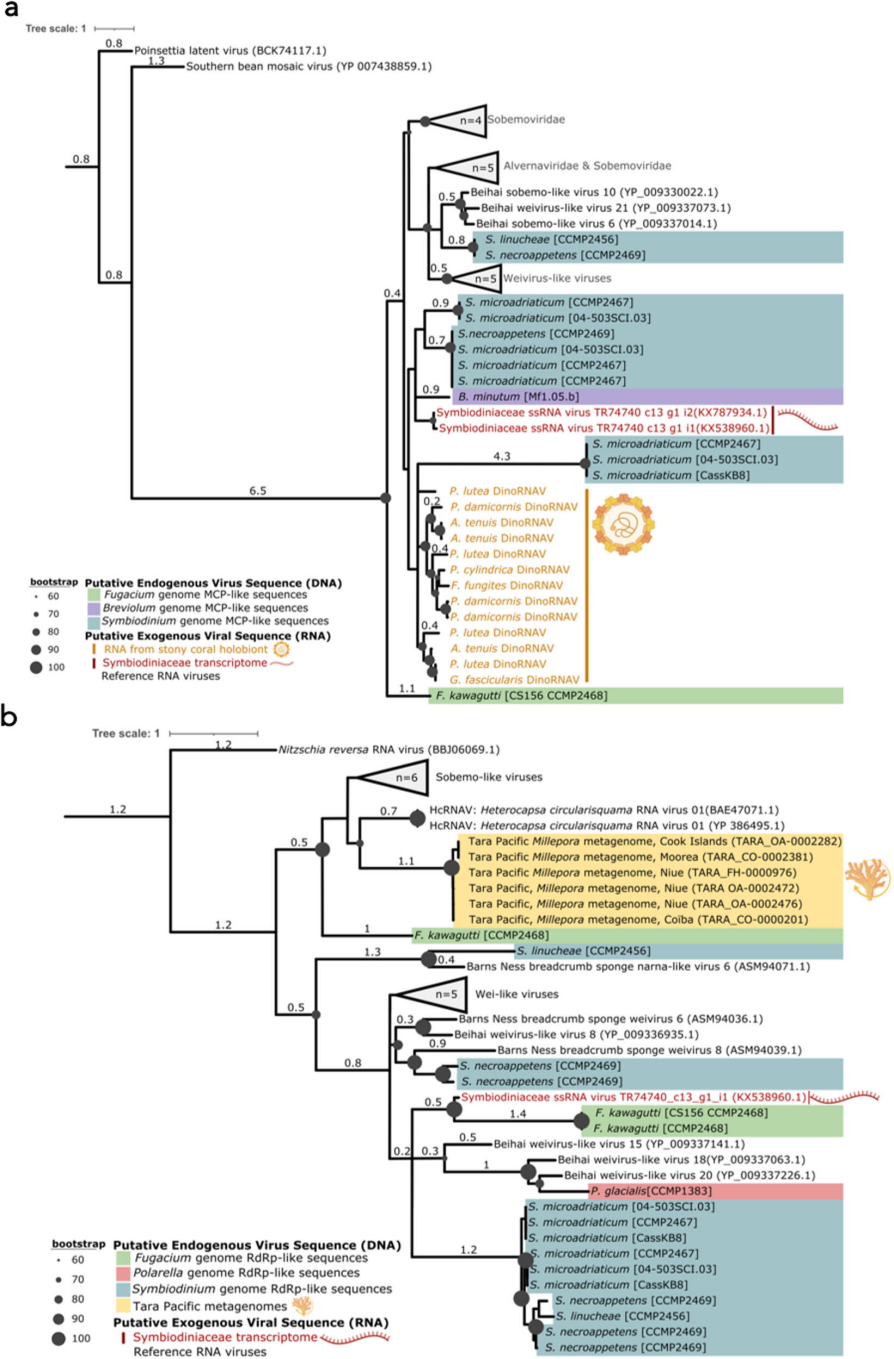
Phylogenies of dinoRNAV major capsid protein (MCP) and RNA-dependent RNA polymerase (RdRp) ORFs. **a** dinoRNAV Major Capsid Protein (MCP) ORF phylogeny. Maximum-likelihood tree of MCP amino acid sequences generated with a LG + F + G4 substitution model and 50,000 parametric bootstraps, illustrating the similarity of putative dinoRNAV EVEs (this study) to extant dinoRNAVs from stony coral colonies. **b** RNA-dependent RNA polymerase (RdRp) ORF phylogeny. Maximum-likelihood tree of RdRp amino acid sequences generated with a Blosum62 + G4 substitution model and 50,000 parametric bootstraps, demonstrating the similarity of metagenomic dinoRNAV EVE RdRps to RdRps of the sole recognized *Dinornavirus*, *Heterocapsa circularisquama* RNA virus (HcRNAV), as well as alignment to each other. ORFs were recovered from host metagenomes, transcriptomes, genomes, and extant +ssRNA reference viruses from amplicon libraries (a only). Both trees include *Dinornavirus* reference sequences and visualized in iTOL.

Modern, exogenous dinoRNAVs (Order: *Sobelivirales*) are highly divergent and hypothesized to form chronic infections within dinoflagellate hosts^54,55,57,58^. This chronic infection strategy likely provides opportunities for retroelement-driven endogenization into host genomes. Because many EVEs evolve at the rate of the host genome, rather than at the much faster rate of exogenous +ssRNA viral genomes, EVEs can serve as a snapshot of viral ancestry^90^. We compared translated dinoRNAV EVEs to exogenous dinoRNAVs and other *Dinornavirus* taxa to assess the conservation of EVEs, the potential for host utilization of these elements, and their relatedness to contemporary dinoRNAVs. We found that amino acid translations of endogenous dinoRNAV *MCP* sequences contained conserved motifs observed in the exogenous *MCP* sequences (e.g. Regions 1–3 in Fig. 3), yet the associated phylogeny was highly polyphyletic along inferred ancestral nodes (Fig. 5a). Endogenous *MCP* ORFs also appear to be evolving under neutral selection (dN/dS=0.958).

Endogenized dinoRNAV MCP form their own clades within the MCP tree, each closely related to specific clades consisting of extant dinoRNAVs or environmental (i.e. unclassified) sobeliviruses with similar conserved motifs. The majority of dinoRNAV *MCP* EVEs shared similarity to extant *MCP*s identified from unfractionated stony coral holobionts via amplicon sequencing^58^; these sequences formed an independent, disorganized clade (Fig. 5a clade containing yellow and blue sequences), relative to those recovered from dinoflagellate transcriptomes or those of other invertebrate hosts. Likewise, dinoRNAV *RdRp* EVEs identified via metagenomics appear most similar to HcRNAV, the defining member of family Alvernaviridae and a protist pathogen, further supporting the affiliation of this EVE with a dinoflagellate host. *MCP* and *RdRp* ORFs putatively derived from the same dinoflagellate genomes often shared clades (clades containing multiple blue or green sequences in Fig. 5a, b), perhaps indicative of duplications within genomes or multiple integration events of particular dinoRNAV lineages within host genera. The detection of putative dinoRNAV *RdRp* ORFs within *Polarella* genomes is therefore indicative of either the antiquity of dinoRNAV-dinoflagellate interactions and/or a propensity for recent dinoRNAV integration across Dinophyceae families. However, the exclusion of the *P. glacialis* dinoRNAV-*RdRp* from *RdRps* of other dinoflagellate clades (pink, Fig. 5B) further illustrates the congruence between EVEs and their host genomes. Overall, the evident homology to contemporary *Dinornaviruses* support these integrations as Alvernaviridae within order *Sobelivirales*.

The expression and functional potential of endogenized dinoRNAV elements (if any) remains unclear. With no isolated Symbiodiniaceae-infecting dinoRNAV strains available, investigation into EVE functionality is limited to in silico approaches. Sequence data mining efforts identified RNA sequences either sharing sequence similarity with dinoRNAVs, or containing whole dinoRNAV-like ORFs that also annotated as dinoflagellate transcripts (i.e. with cellular ORFs or sequence similarity) in seven out of nine publicly accessible dinoflagellate transcriptomes (Supplementary Data 13). Additionally, two transcripts from an exogenous dinoRNAV infection identified in *Cladocopium* transcriptomes carried *MCP* ORFs of +ssRNA viral sequences (‘TR74740_c13-g1_i1’ and ‘TR74740_c13-g1_i2’^57^, red text in Fig. 5a) and form a clade with putative *Symbiodinium* dinoRNAV EVEs (Fig. 5). Likewise, the *RdRp* ORF of ‘TR74740_c13-g1_i1’ and the *RdRp* of ‘GAKY01194223.1’— a transcript derived from a cultured *Symbiodinium microadriaticum* A1 transcriptome—shared some areas of similarity to putative endogenous dinoRNAVs (Fig. 5b^57,91^. Importantly, both RNA transcripts also shared features characteristic of dinoflagellates, such as a 5’ dinoSL^61^ or dinoflagellate sequence space flanking the dinoRNAV itself^91^. Furthermore, ‘TR74740_c13-g1_i1’ appeared to be in the top 0.03% of expressed transcripts at under certain thermal conditions, and GAKY01194223.1 appeared to exhibit moderately differential expression at the extremes of temperature and ionic stress in a cultured host^57,91^.

While viral *RdRps* have been leveraged by eukaryotes in multiple pathways^92^, the apparent fragmentation of the putative dinoRNAV EVEs in silico may indicate a role in triggering antiviral mechanisms within their hosts^31,93^. Given that the *Symbiodinium* genome contains all core RNAi protein machinery, including Argonaute and Dicer, and that GAKY01194223.1 folds into several hairpins (ΔG = −142.5 kcal/mol; Supplementary Fig. 5 examples), Symbiodiniaceae may use the putative EVE ncRNA identified here to develop host immunity against extant, exogenous dinoRNAVs. Furthermore, Symbiodiniaceae harboring dinoRNAV EVEs also contained numerous non-retroviral EVEs of other viral families (Supplementary Data 11, Fig. 7) in close proximity, such as *Herpesviridae, Baculoviridae, Poxviridae, Iridoviridae, Phycodnaviridae, Pandoraviridae* and *Pithoviridae*, ssDNA viruses of the family *Shotokuvirae*, -ssRNA viruses from the family *Rhabdoviridae* and +ssRNA viruses from the family *Coronaviridae* (Supplementary Fig. 6). Metagenomes corroborate findings of similar *RdRps* from these viral families (Supplementary Fig. 6). This provides support for host-mediated integration (e.g. retroposition) as a means of defense for single celled organisms, though further research is needed^94^.

### Conclusions

Over recent decades, endogenous viral elements (EVEs) have enabled investigators to better understand the evolutionary history of viruses (“paleovirology”) in diverse terrestrial systems, uncovering ancient and modern virus-host interactions. Our study further demonstrates how in silico identification of EVEs can provide ecological context for enigmatic viral genomes in non-model, multipartite systems such as coral holobionts, impacting how we study coral reefs and their viral consortia. Here, we detected heritable integrations of multiple putative dinoRNAV genes in Symbiodiniaceae scaffolds from cnidarian metagenomes, as well as in diverse genomes of cultured Symbiodiniaceae; no integrations were detected from seawater metagenomes nor diverse aposymbiotic cnidarian genomes. The apparent pervasive nature of dinoRNAV-like sequences among dinoflagellate genomes (especially the genus *Symbiodinium*) suggests widespread and recurrent/ancestral integration of these EVEs. We propose that host-provisioned mechanisms drive dinoRNAV integration into single-celled dinoflagellate genomes as EVEs. The findings presented in this study further validate the dinoRNAV-Symbiodiniaceae virus-host pair, enhancing our understanding of ecologically and economically important cnidarian holobionts and opening the door to examining the role of EVEs in reef health.

## Methods

### Identification and computational validation of dinoRNAV EVEs leveraging meta’omics

The Tara Pacific Expedition (2016-2018) sampled coral reefs to investigate reef health and ecology using multiple methods, including amplicon sequencing and metagenomics (see Pesant et al. 2020^95^ and 10.5281/zenodo.4068293 for coral reef sampling and processing methods). In this study, we explored metagenomes generated from hydrocorals (*n* = 60 *Millepora*), stony corals (*n* = 108 *Porites*, *n* = 101 *Pocillopora*) sampled from 11 islands (three replicate sites per island) across the South Pacific Ocean during the Tara Pacific Expedition for dinoRNAV EVEs (Fig. 1, Supplementary Data 1A, 1B^95^). Amplicon libraries of the dinoflagellate Internal Transcribed Spacer 2 (ITS2) gene fragment were sequenced in tandem with the metagenomes, to characterize the dominant Symbiodiniaceae harbored by hydrozoan and stony coral colonies^95^.

To confirm that these dinoRNAV EVE sequences were affiliated with coral holobionts and reduce the possibility that they are technical artifacts, publicly available metagenome libraries were analyzed (Supplementary Data 1B). These additional libraries included 120 assembled pelagic water samples presumed to include pelagic dinoflagellate sequences from the Tara Oceans dataset (2009-2013^70^) and 30 MiSeq metagenomes from unfractionated samples of the stony coral genus *Acropora*, which were processed and sequenced via a different pipeline (Supplementary Data 1B, Supplementary Fig. 7). Publicly accessible transcriptomes from nine Symbiodiniaceae assemblies (Supplementary Data 1B) were also queried to determine if dinoRNAV-like sequences were present in poly(A)-selected dinoflagellate transcriptomes and resembled EVEs in terms of proximal gene composition and presence of a characteristic pre-mRNA spliced leader (dinoSL) sequence (as in Levin et al, 2017^57^). Details regarding the collection of samples, generation of metagenomes and associated Symbiodiniaceae amplicon libraries, and associated bioinformatic analyses are provided in Supplementary Fig. 7).

Metagenomic and transcriptomic scaffolds were annotated against a curated database of dinoRNAV-like sequences (Supplementary Data 14) via BLASTx (e-value < 1 × 10^−5^; see Supplementary Fig. 7 for workflow^96^). Alignments to the custom database with a bit score <50 and percent shared amino acid identity <30% were excluded from further analysis. A length penalty was not imposed during this step due to the limited length of assembled scaffolds (average N50 = 3341 ± 127 nt across all queried libraries). Open reading frames (ORFs) from selected scaffolds were called via Prodigal (v.2.6.3^97^) and annotated against the NCBI-nr database (e-value < 0.001; DIAMOND v.2.0.6^98^) to confirm homology to dinoRNAVs and to identify adjacent dinoflagellate sequences (e-value < 1 × 10^-5^, bit*≥*50). In the absence of complete ORFs (potentially due to the limited size of scaffolds, partial integrations, etc.), homology was confirmed through comparison of the initial alignments to the curated database and 300nt of upstream/downstream flanking sequences (bedtools v.2.30.0^99^) against the NCBI-nr database (e-value < 0.001; DIAMOND v.2.0.6^98^). This served as further curation and verification, as EVEs can exist in fragmented or degraded states. Non-normalized quality-controlled reads were mapped via bbmap (v.38.84^100^), and putative EVEs were assessed for uniform read coverage across scaffolds, reducing the probability of chimeric assembly. RNA secondary structure was predicted via mfold (v.3.5^101^).

### dinoRNAV EVEs in dinoflagellate and aposymbiotic cnidarian genomes

Publicly available dinoflagellate and aposymbiotic (dinoflagellate-free) cnidarian genome assemblies were queried to resolve the putative host(s) of dinoRNAVs, to assess homology among detected dinoRNAVs within coral holobionts, and to compare genes proximal to dinoRNAV EVEs in different host species/strains. A chromosome-scale dinoflagellate genome assembly generated from a *Symbiodinium microadriaticum* culture (Accession: GSE152150)^82^, and scaffold-scale genome assemblies were examined for dinoRNAV EVEs (Supplementary Data 1B, Supplementary Fig. 7). Scaffold-scale genome assemblies were from the closely related families Symbiodiniaceae and Suessiaceae, and included representatives from the genera *Symbiodinium* (*n* = 9), *Breviolum* (*n* = 1), *Cladocopium* (*n* = 3), *Durusdinium* (*n* = 1), *Fugacium* (*n* = 2), and *Polarella* (*n* = 2), as well as 25 aposymbiotic cnidarian genome assemblies, including the stony coral genera *Acropora* (*n* = 13), *Astreopora* (*n* = 1), *Galaxea* (*n* = 1), *Montastraea* (*n* = 1), *Montipora* (*n* = 3), *Orbicella* (*n* = 1), *Pocillopora* (*n* = 2), *Porites* (*n* = 1), and *Stylophora* (*n* = 1), and the jellyfish *Clytia* (*n* = 1; Fig. 2, Supplementary Data 1B). All publicly available genome assemblies had undergone a form of microbial decontamination, trimming, and quality control prior to assembly, minimizing risk of microbial contamination. Genome completeness and quality further were assessed via BUSCO (v3)^102^ with the Eukaryota dataset and QUAST (v5.0.2^103^), respectively. Scaffolds/chromosomes containing putative dinoRNAV EVEs were identified by aligning sequences to the protein version of the Reference Viral DataBase (RVDB v.19^104^) using DIAMOND BLASTx (v0.9.30)^98^. The same exclusion criteria were maintained for alignments of metagenomic scaffolds, also omitting alignments <100 amino acids. Regions of dinoflagellate genomes exhibiting similarity to the *MCP* or *RdRp* of reef-associated dinoRNAV reference genomes^57^ or other closely related +ssRNA viruses (Supplementary Data 14) were extracted and re-aligned to the NCBI-nr database to further confirm viral homology.

We tested the relationship between the number of identified dinoRNAV EVE-containing scaffolds, dinoflagellate genera, and genome quality metrics using a linear model. Model selection was performed with an F-test (package car, v.3.0-12) and assumptions were visually checked. Pairwise comparisons between genera were conducted using the package emmeans (v.1.7.2). Putative whole dinoRNAV-like genomes within scaffolds were identified based on the presence of *MCP* and *RdRp-*like sequences on the same scaffold no further than 1.5 Kbp apart (Table 1; Supplementary Fig. 3). IRESPred^105^ was utilized to identify internal ribosomal entry sites (IRES) with default parameters on putative dinoRNAV EVE with whole sequence integrations.

ORFs were predicted and annotated from dinoRNAV EVE-containing scaffolds and all dinoflagellate chromosomes using Prodigal^97^ and MAKER2 annotation pipeline^106^ with the AUGUSTUS gene prediction software^107^. Translated ORFs were then aligned to a hybrid database containing the UniProt/Swiss-Prot database and protein version of RVDB (v.19; DIAMOND-BLASTp). ORFs on putative dinoRNAV EVE-containing scaffolds and chromosomes were further annotated using InterProScan (v5.48-83.0, Pfam analysis with default parameters) to identify sequences proximal to putative dinoRNAV integrations. The presence of dinoflagellate spliced leaders (“dinoSLs”) were examined within 500nt of dinoRNAV EVEs using BLASTn with default parameters (except word size=9, excluding two ambiguous positions as specified in Gonzalez-Pech et al. 2021^83^).

### Phylogenetic analysis of dinoRNAV EVEs

Amino acid-based phylogenetic trees were generated with dinoRNAV EVE ORFs (MCP and RdRp) from scaffold-scale genomic assemblies, metagenomes, transcriptomes, and sequences from exogenous and closely related +ssRNA reference viruses (Supplementary Data 1 A, B, Supplementary Data 14). Sequences were aligned using the best fit algorithm determined by MAFFT (v7.464)^108^ and reviewed and trimmed manually in MEGA (v7)^109^. Maximum-likelihood trees were generated with IQTREE2^110^ using the model determined by ModelFinder^111^ and 50,000 parametric bootstraps^112^ with nearest neighbor interchange optimization. ORFs from the chromosome-level assembly for S. microadriaticum culture CCMP2467 were not included in the phylogeny in order to avoid redundancy with those from the analogous scaffold-level assembly. To calculate dN/dS, ORFs were aligned in Clustal Omega (v.1.2.4), refined in MUSCLE (v.3.6), before using pal2nal (v.14) for codon-based nucleic acid alignment. Evolutionary trajectory was then assessed via CODEML (PAML package, v.4.10.5).

### Statistics and reproducibility

As indicated throughout the article, metagenomes (*n* = 269) and genomes (*n* = 18) served as technical replicates, and ORFs or full EVE sequences served as comparative ecoevolutionary units (replicates described in Table 1) when available. Negative controls (seawater metagenomes, coral host, etc) were also evaluated. All statistical packages are reported in methods or Supplementary Fig. 7.

### Reporting summary

Further information on research design and collection permits are available in the Nature Research Reporting Summary linked to this article.

## Supplementary information

Supplementary Information

Description of Additional Supplementary Files

Supplementary Data

Reporting Summary

## Data Availability

Metadata are accessible in zenodo: https://zenodo.org/record/6299409#.Y-ClwuzMKml. Metagenomes are available via 10.5281/zenodo.7839794. Seawater metagenomes are available through the European bioinformatics institute (Tara Oceans; ERP001736) and NCBI (PRJEB1787). NCBI accession numbers for individual holobiont species metagenomes, genome assemblies and reference sequences can be found in Supplementary Data 1B, 3 and 14, respectively.
