# Supplementary material for: Endogenous viral elements reveal associations between a non-retroviral RNA virus and symbiotic dinoflagellate genomes
Source: Commun Biol. 2023 Jun 1;6:566. doi: 10.1038/s42003-023-04917-9 (PMC10235124; doi:10.1038/s42003-023-04917-9)
Supplement: Supplementary file 1 — Supplementary Information [file 42003_2023_4917_MOESM1_ESM.pdf]

Supplementary Figures - *Endogenous viral elements reveal associations between a non-retroviral RNA virus and symbiotic dinoflagellate genomes*

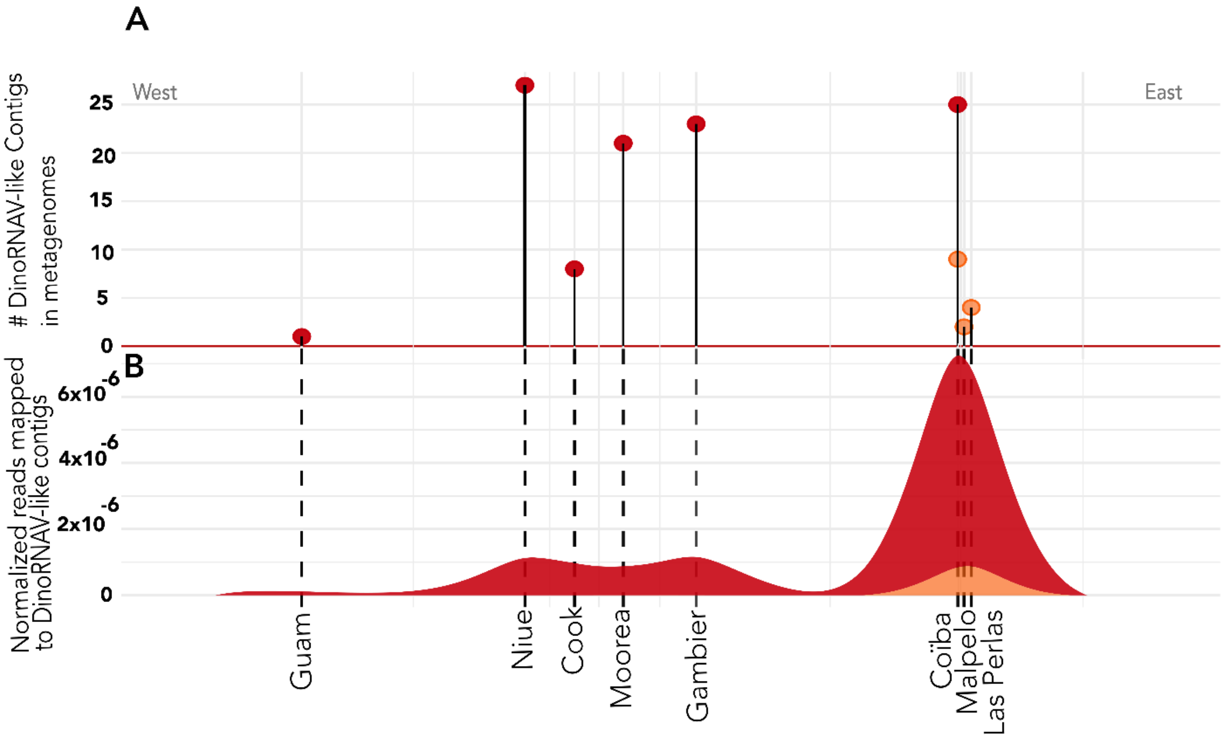

Supplementary Figure 1. Islands correlating with dinoRNAV EVE-like sequence detection among Tara Pacific metagenomes indicating reads mapped to dinoRNAV-like scaffolds within individual Tara Pacific metagenomic libraries, normalized by quality-controlled reads. See Supplementary Data 2 for supporting data (total n=120 contigs)

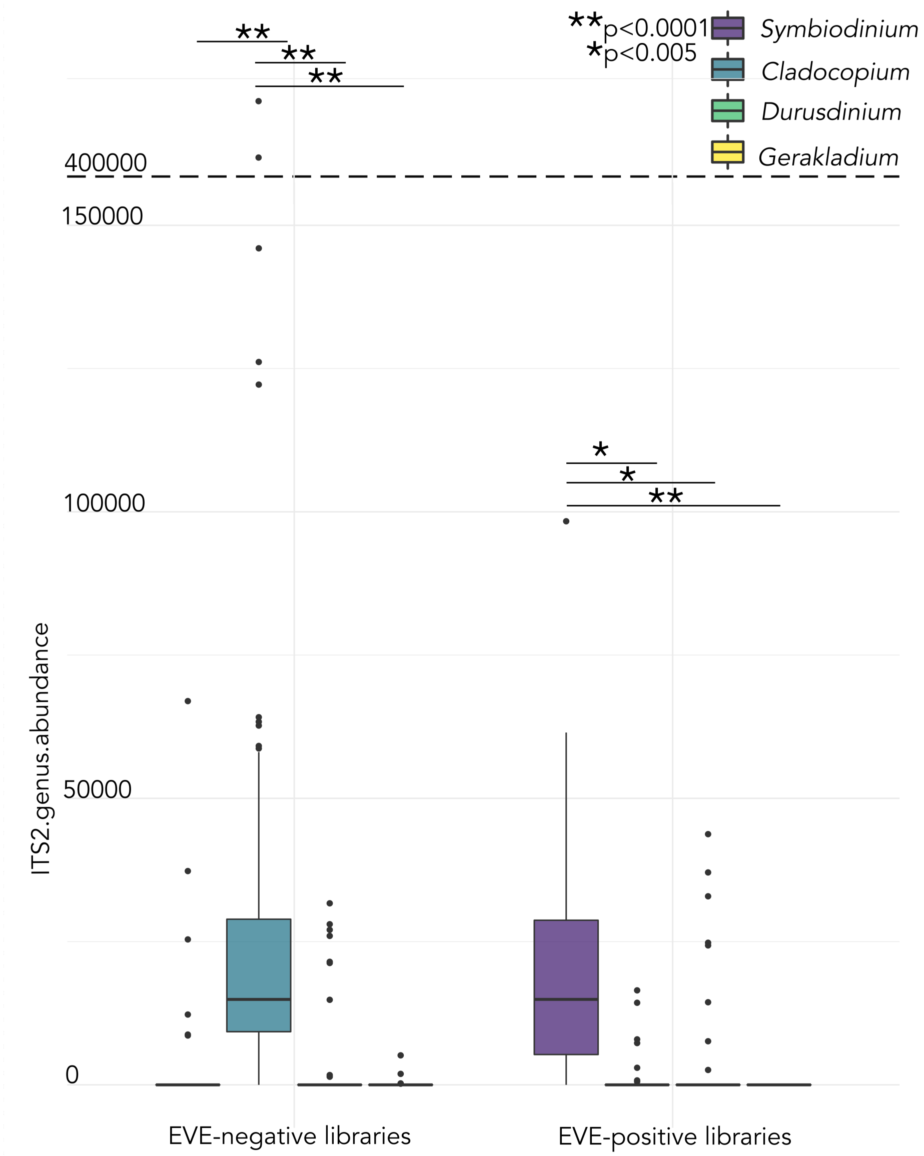

Supplementary Figure 2. DinoRNAV EVE detection - regardless of host species - largely correlated with *Symbiodinium* dominated communities. $F_{2,1044}$=25.8, p<0.0001, Type III ANOVA with Satterthwaite's. *Posthoc*: pairwise Tukey with Kenward-Roger approximation for linear mixed models (replicate libraries were randomized and nested within islands and examined if EVE-positive libraries (factor 1) are affiliated with genus (factor 2) with median and 95% CI error bars (n=263 unique metagenomes) . See Supplementary Data 5 for supporting data.

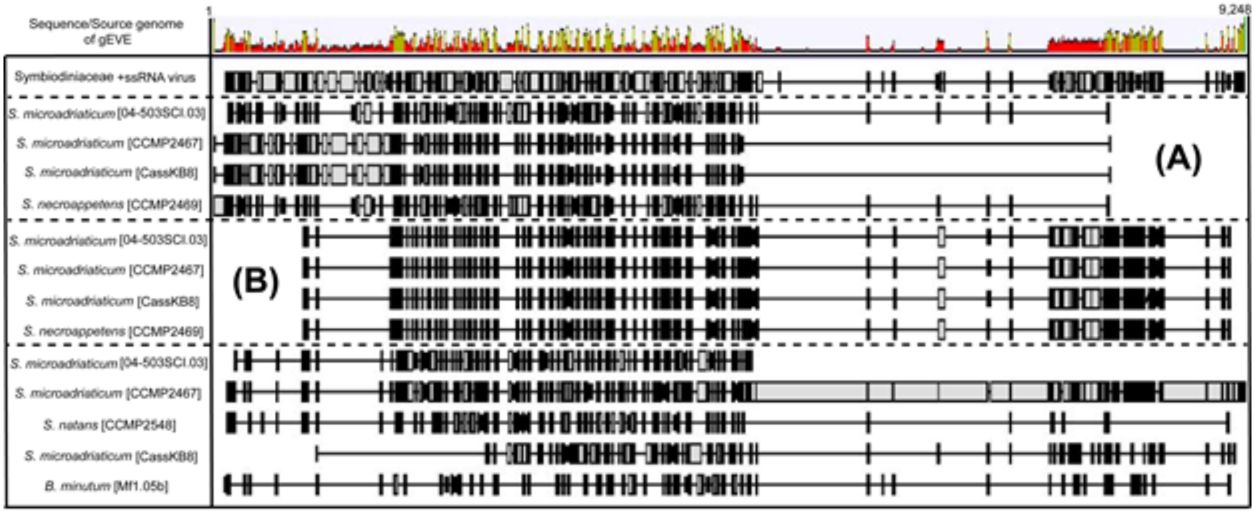

Supplementary Figure 3. Alignment of putative endogenous genomic dinoRNAV EVEs to the Symbiodiniaceae-infecting +ssRNA virus genome assembled by Levin et al (2017)[57] from a *Symbiodinium* spp. transcriptome. Repeated "whole" genome EVEs (A & B) showing similarity across *Symbiodinium* genome are marked with dashed boxes. Putative whole genome EVEs were extracted from their respective scaffolds and aligned to the dinoRNAV reference genomes using MAFFT (v7.464, Katoh and Standley 2013) and visualized in Geneious Prime (v.2021.0.1, restricted).

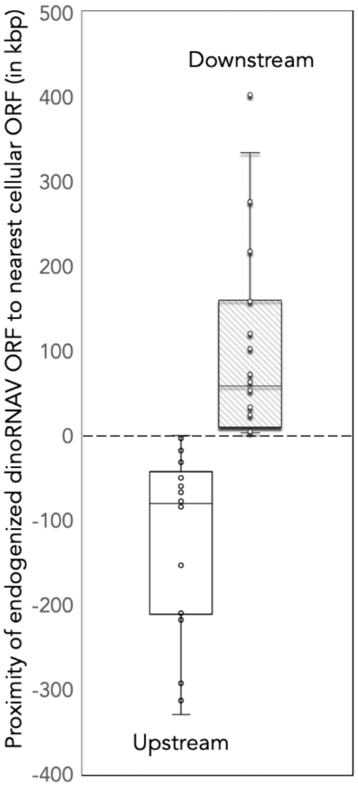

Supplementary Figure 4. Box and whisker plot indicative of median and quartiles of nearest 3’ or 5’ proximal genes (in bp; whiskers are 95% CI; n=22 integrations on n=18 chromosomes) to dinoRNAV EVEs on the chromosome level *Symbiodinium microadriaticum* assembly (Smic1.1N, GSE152150).

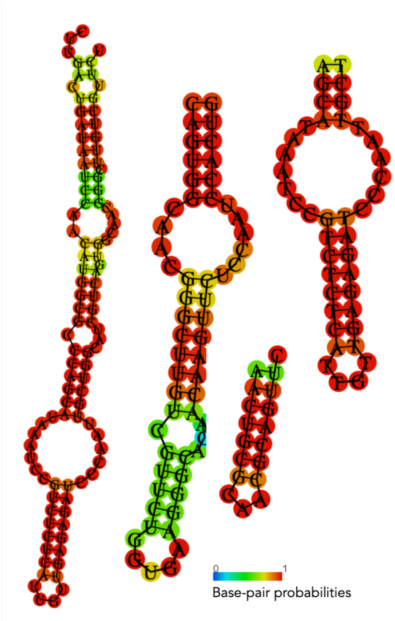

Supplementary Figure 5. Example secondary structural elements of *Symbiodinium* sp. (A1) transcript Locus_83084 (accn: GAKY01194223.1) including hairpin folds. *Symbiodinium* genome contains all core RNAi protein machinery, including Argonaute and Dicer and GAKY01194223.1 folds into several hairpins (ΔG = -142.5kcal/mol; mfold v. 3.5)

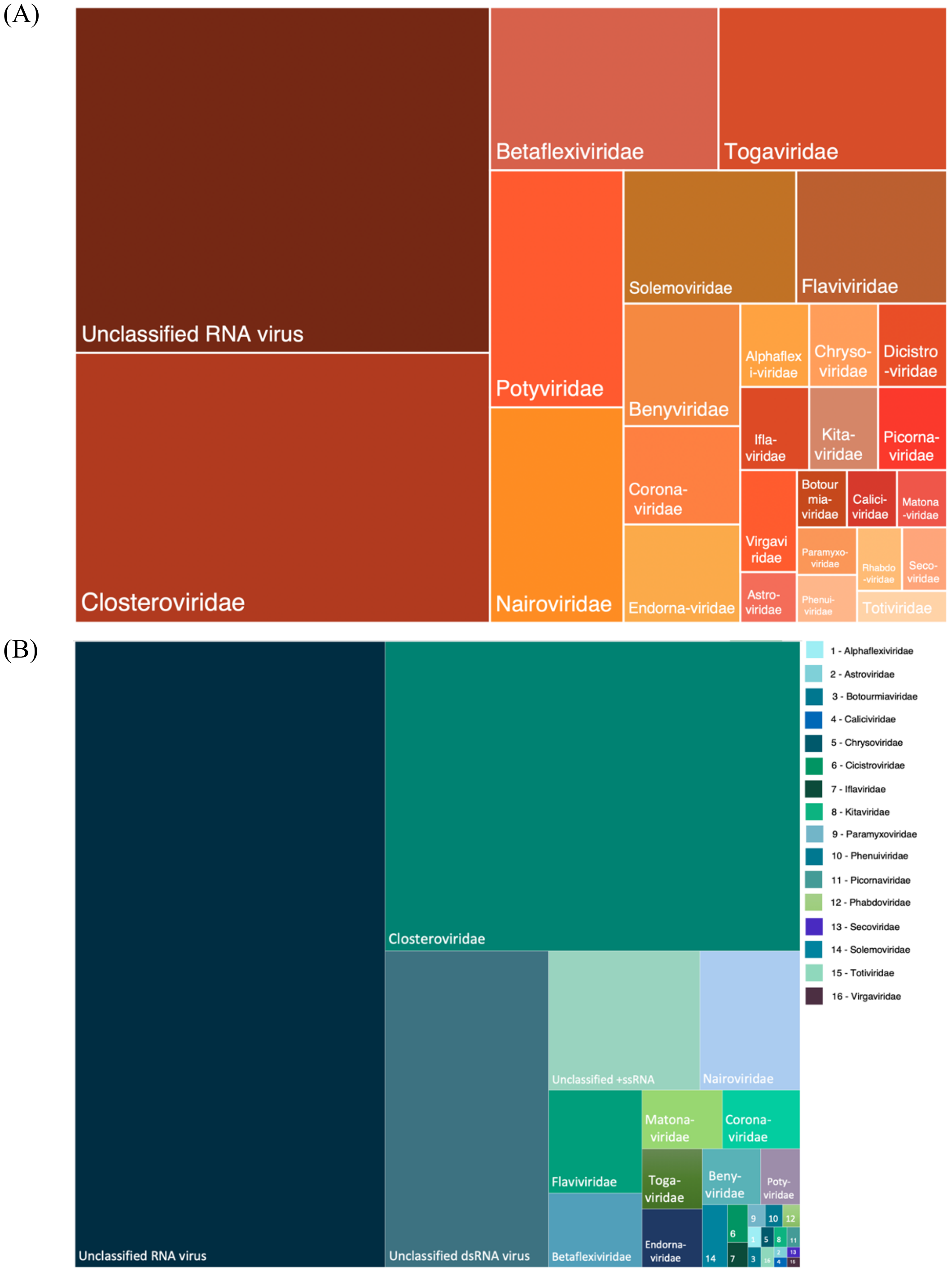

Supplemental Figure 6. Composition of RNA virus families detected in Tara Pacific metagenomes where DinoRNAV EVE was also detected per identification and characterization of *RdRp* gene via Diamond BLASTx. (A) Map describes the composition of *unique* contigs affiliated with each RNA viral family (per *RdRp* detection) across 42 dinoRNAV EVE-positive metagenomes (representing n=26 families and 50 RNA viral contigs containing *RdRps* with unknown viral families; total of n=187 *unique* contigs); (B) Map describes the *total* number of contigs identified within each RNA viral family across 42 dinoRNAV EVE-positive metagenomes (per *RdRp* detection; n=3,5069 *total* contigs identified; average 834.97 per library).

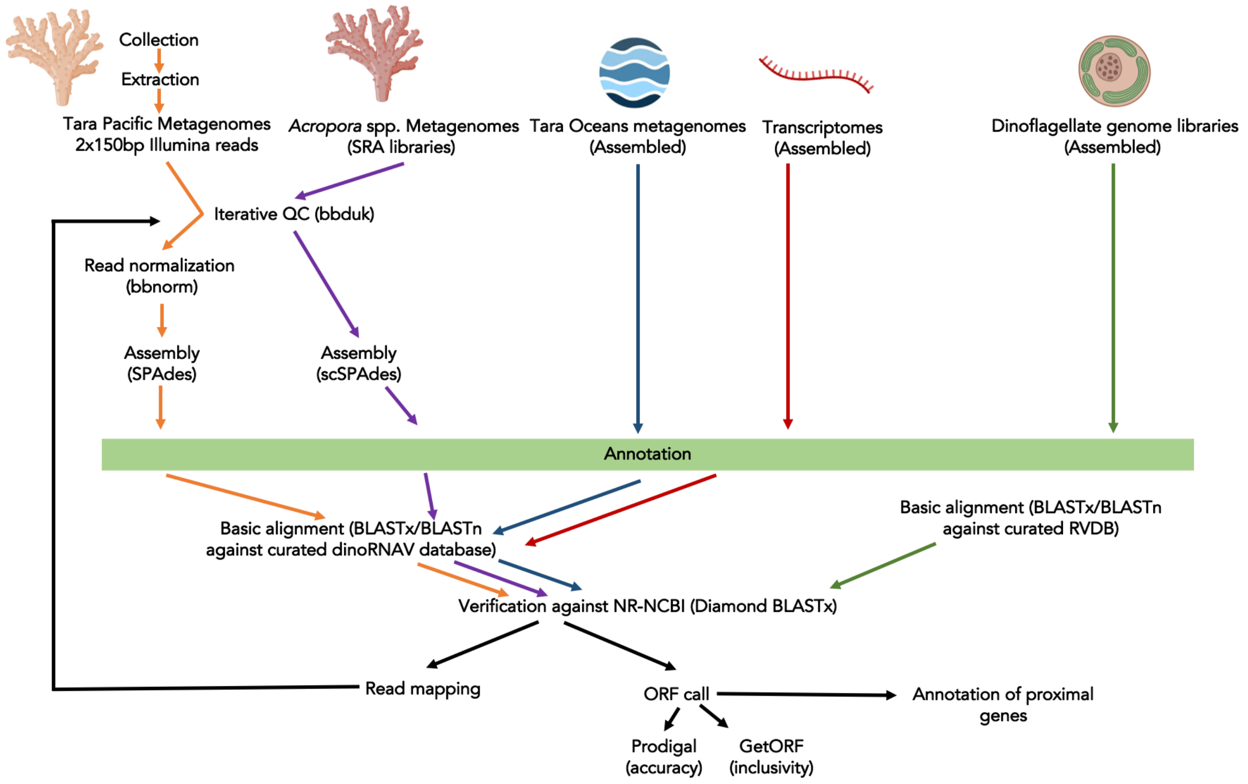

Supplementary Fig 7. Acquisition and bioinformatic analysis pipeline utilized for dinoRNAV EVE identification and verification. Five sources of data (newly assembled metagenomes from Tara Pacific, newly assembled metagenomes from publicly available *Acropora* holobionts, pre-assembled metagenomes from Tara Ocean databases, pre-assembled transcriptomes from Baumgarten et al, 2020[101], and publicly available genomes from dinoflagellates) were quality controlled/assembled per a variety of software in the bbtools suite and SPAdes prior to alignment-based annotation against several databases (NR-NCBI, UniProt, RVDB, or manually curated databases of dinoRNAV genomes. Details not available in “Methods” can be found below:

Collection and sequencing of Tara Pacific coral metagenomes (see full methods in doi:10.5281/zenodo.4068293):   Briefly, coral nubbins were collected from replicate colonies of a fire coral (genus: *Millepora*, n=60), and two stony corals (genera: *Porites*, n=108; and *Pocillopora*, n=101), as well as from immediately surrounding water. DNA was extracted via Quick-DNA/RNA Kit with supplemental enzymatic lysis (Zymo Research, Irvine, CA, USA). mechanically fragmented (300bp; Covaris E210, Covaris, Inc., USA), end-repaired and polyadenylated (NEBNext DNA Modules; New England Biolabs, MA, USA) before being ligated to adapters (NextFlex DNA barcodes; Bio Scientific Co) for clean-up and amplification. DNA was subjected to 2x150bp paired-end Illumina sequencing (Genoscope; Évry, France). Metagenomic libraries were trimmed for length, quality, and adapters (bbduk v.38.06), normalized (bbnorm v. 38.06; target depth 40x coverage) and assembled *de novo* (SPAdes v.3.12).

Publicly available metagenomes were quality controlled as detailed above, non-normalized, and assembled via SPAdes utilizing the single cell modifier (v.3.12).

Collection and sequencing of Tara Pacific coral amplicon libraries (see full methods in doi:10.5281/zenodo.4061797 and Belser et al, *in prep*): DNA was extracted using the Quick-DNA/RNA kit with supplemental enzymatic lysis (Zymo Research, Irvine, CA, USA), PCR amplified in triplicate using SYM-VAR-5.8S2 / SYM-VAR-REV (Hume et al. 2019), and cleaned (AMPure XP; Beckman Coulter, Brea, CA) prior to pooling and library construction (NEBNext DNA Modules; New England

Biolabs, MA, USA). Amplicons were sequenced on the Illumina platform. "Defining intragenomic variants" were utilized to differentiate taxonomic profiles within the Symportal analytical framework post-minimum entropy decomposition (Hume et al, 2019)[111].

Transcriptomes: To assess (1) if dinoRNAV EVEs were present in poly(A)-selected dinoflagellate transcriptomes, and (2) if these transcripts resembled endogenized dinoRNAVs in proximal gene composition, presence of a characteristic pre-mRNA spliced leader (SL) sequence (SL; as in Levin et al, 2017), or were differentially expressed under variable environmental conditions. Publicly accessible transcriptomes from the genus *Symbiodinium* (n=11 smRNA, n=11 mRNA; Supplemental Table ST.1B) were queried for dinoRNAV-like sequences. Reads were trimmed and quality controlled per the same description as the original study (cutadapt v. 3.1; Baumgarten et al, 2020). Pre-assembled RNA sequences from nine publicly available transcriptomes (Supplemental Data 1) were congruently queried. Transcripts sharing sequence similarity with dinoRNAVs, or containing whole dinoRNAV-like ORFs, and simultaneously annotating as dinoflagellate transcripts (i.e. with cellular ORFs or sequence similarity) were manually annotated. Transcripts were annotated for dinoRNAVs in congruent manner as metagenomes (described in Methods), reads were mapped via bbmap (v.38.84), and RNA secondary structure was predicted via mfold (v.3.5).

Members of Tara Consortium who coordinated Tara Pacific collection and study efforts

Sylvain Agostini (orcid.org/0000-0001-9040-9296) Shimoda Marine Research Center, University of Tsukuba, 5-10-1, Shimoda, Shizuoka, Japan
Denis Allemand (orcid.org/0000-0002-3089-4290) Centre Scientifique de Monaco, 8 Quai Antoine Ier, MC-98000, Principality of Monaco
Bernard Banaigs (orcid.org/0000-0003-3473-4283) PSL Research University: EPHE-UPVD-CNRS, USR 3278 CRIOBE, Université de Perpignan, France
Emilie Boissin (orcid.org/0000-0002-4110-790X) PSL Research University: EPHE-UPVD-CNRS, USR 3278 CRIOBE, Laboratoire d'Excellence CORAIL, Université de Perpignan, 52 Avenue Paul Alduy, 66860 Perpignan Cedex, France
Emmanuel Boss (orcid.org/0000-0002-8334-9595) School of Marine Sciences, University of Maine, Orono, 04469, Maine, USA
Chris Bowler (orcid.org/0000-0003-3835-6187) Institut de Biologie de l'Ecole Normale Supérieure (IBENS), Ecole normale supérieure, CNRS, INSERM, Université PSL, 75005 Paris, France
Colomban de Vargas (orcid.org/0000-0002-6476-6019) Sorbonne Université, CNRS, Station Biologique de Roscoff, AD2M, UMR 7144, ECOMAP 29680 Roscoff, France & Research Federation for the study of Global Ocean Systems Ecology and Evolution, FR2022/ Tara Oceans-GOSEE, 3 rue Michel-Ange, 75016 Paris, France
Eric Douville (orcid.org/0000-0002-6673-1768) Laboratoire des Sciences du Climat et de l'Environnement, LSCE/IPSL, CEA-CNRS-UVSQ, Université Paris-Saclay, F-91191 Gif-sur-Yvette, France
Michel Flores (orcid.org/0000-0003-3609-286X) Weizmann Institute of Science, Department of Earth and Planetary Sciences, 76100 Rehovot, Israel
Didier Forcioli (orcid.org/0000-0002-5505-0932) Université Côte d'Azur, CNRS, INSERM, IRCAN, Medical School, Nice, France and Department of Medical Genetics, CHU of Nice, France
Paola Furla (orcid.org/0000-0001-9899-942X) Université Côte d'Azur, CNRS, INSERM, IRCAN, Medical School, Nice, France and Department of Medical Genetics, CHU of Nice, France
Pierre Galand (orcid.org/0000-0002-2238-3247) Sorbonne Université, CNRS, Laboratoire d'Ecogéochimie des Environnements Benthiques (LECOB), Observatoire Océanologique de Banyuls, 66650 Banyuls sur mer, France
Eric Gilson (orcid.org/0000-0001-5738-6723) Université Côte d'Azur, CNRS, Inserm, IRCAN, France
Fabien Lombard (orcid.org/0000-0002-8626-8782) Sorbonne Université, Institut de la Mer de Villefranche sur mer, Laboratoire d'Océanographie de Villefranche, F-06230 Villefranche-sur-Mer, France
Stéphane Pesant (orcid.org/0000-0002-4936-5209) European Molecular Biology Laboratory, European Bioinformatics Institute, Wellcome Genome Campus, Hinxton, Cambridge CB10 1SD, UK
Serge Planes (orcid.org/0000-0002-5689-5371) PSL Research University: EPHE-UPVD-CNRS, USR 3278 CRIOBE, Laboratoire d'Excellence CORAIL, Université de Perpignan, 52 Avenue Paul Alduy, 66860 Perpignan Cedex, France
Stéphanie Reynaud (orcid.org/0000-0001-9975-6075) Centre Scientifique de Monaco, 8 Quai Antoine Ier, MC-98000, Principality of Monaco
Matthew B. Sullivan (orcid.org/0000-0003-4040-9831) The Ohio State University, Departments of Microbiology and Civil, Environmental and Geodetic Engineering, Columbus, Ohio, 43210 USA
Shinichi Sunagawa (orcid.org/0000-0003-3065-0314) Department of Biology, Institute of Microbiology and Swiss Institute of Bioinformatics, Vladimir-Prelog-Weg 4, ETH Zürich, CH-8093 Zürich, Switzerland
Olivier Thomas (orcid.org/0000-0002-5708-1409) Marine Biodiscovery Laboratory, School of Chemistry and Ryan Institute, National University of Ireland, Galway, Ireland

Romain Troublé (ORCID not-available) Fondation Tara Océan, Base Tara, 8 rue de Prague, 75 012 Paris, France
Rebecca Vega Thurber (orcid.org/0000-0003-3516-2061) Oregon State University, Department of Microbiology, 220 Nash Hall, 97331Corvallis OR USA
Christian R. Voolstra (orcid.org/0000-0003-4555-3795) Department of Biology, University of Konstanz, 78457 Konstanz, Germany
Patrick Wincker (orcid.org/0000-0001-7562-3454) Génomique Métabolique, Genoscope, Institut François Jacob, CEA, CNRS, Univ Evry, Université Paris-Saclay, 91057 Evry, France
Didier Zoccola (orcid.org/0000-0002-1524-8098) Centre Scientifique de Monaco, 8 Quai Antoine Ier, MC-98000, Principality of Monaco
