# Supplementary material for: Endogenous viral elements reveal associations between a non-retroviral RNA virus and symbiotic dinoflagellate genomes
Source: Commun Biol. 2023 Jun 1;6:566. doi: 10.1038/s42003-023-04917-9 (PMC10235124; doi:10.1038/s42003-023-04917-9)
Supplement: Supplementary file 2 — Description of Additional Supplementary Files [file 42003_2023_4917_MOESM2_ESM.pdf]

Description of Additional Supplementary Files

File title: Supplementary Data 1A
Description: Metadata for metagenomic libraries of coral and water queried for dinoRNAV EVEs indicating sequencing depth, quality control, and assembly statistics. Table also includes metagenome species (with taxonomic ID) and location (site and colony) per library. Also find a Google Earth map of dinoRNAV EVE-positive sites here: https://bit.ly/3ri6m1V and a map of pelagic water sample collection here: http://ocean-microbiome.embl.de/companion.html (Sunagawa et al, 2015)

Table parameters: Sample - unique library name; Location – island and other geographic indicator, Site_Colony - unique coral colony indicator; Sample_material – type of biological material collected; TaxID – NCBI taxonomic indicator of biological material collected; #raw.PE.reads/bases – number of paired end reads or bases sequenced; #QC.PE.reads/bases – number of quality controlled reads or bases; #contigs – number of contigs assembled from quality controlled reads; N50 – average N50 of contigs; positive_dinoRNAV – number of dinoRNAV EVE sequences identified in library

File title: Supplementary Data 1B
Description: Accession numbers, taxonomy, and submitter data (when available) for publicly available genomes, chromosomes, and metagenomes queried for dinoRNAV EVEs. Table includes datasets for coral genomes, dinoflagellate genomes, dinoflagellate chromosomes, *Acropora* spp. metagenomes, pelagic water metagenomes, and Symbiodiniaceae transcriptomes. Starred genomes in 'Refdb.water' are those utilized as query to determine the presence and quantity of Symbiodiniaceae sequences in pelagic seawater samples via DIAMOND. References 112-119.

File title: Supplementary Data 2
Description: Quantification of hydrocoral metagenome assemblies with and without dinoRNAVs. Hydrocoral metagenomes were sequenced at equivalent depth as scleractinian corals and had comparable level of annotation, precluding methodological bias towards dinoRNAV prevalence among these libraries. Also indicates average reads mapped to dinoRNAVs in EVE-positive libraries.

File title: Supplemental Data 3
Description: Most common genes immediately proximal to putative dinoRNAV EVE integrations identified in metagenomes, including accession numbers and name. DinoRNAV EVE open reading frames (ORFs) appeared to be immediately adjacent to ORFs identified as dinoflagellate genes.

File title: Supplementary Data 4
Description: Frequency of ITS2 profiles, partitioned by coral genus (*Millepora* and *Pocillopora*) and island. ITS2 profiles are sorted by clade.

File title: Supplementary Data 5
Description: Comparative frequency of ITS2 clades between metagenomic libraries with and without dinoRNAV EVE-like sequences. N indicates no dinoRNAV-like sequences, while Y indictcates the presence of at least one scaffold that annotated as a dinoRNAV EVE (see Supplementary Figure 7, Methods for annotation parameters). See Supplementary Figure 2 for statistical test of putative host.

File title: Supplementary Data 6
Description: individual count of the occurrence of dinoRNAV EVE-like annotations in metagenomes and genomes, including negative controls (coral host genomes, seawater metagenomes, Acropora spp. metagenomes), in support of Figure 2.

File title: Supplementary Data 7

Description: Accession information and completeness measured in BUSCO (Benchmarking Universal Single-Copy Orthologs) scores of dinoflagellate genomes queried for dinoRNAV EVEs. While a linear model suggested that there was no relationship between dinoRNAV detection and genome completeness, greater genome completeness could lend more opportunities for dinoRNAV EVE detection.

File title: Supplemental Data 8
Description: Results from a linear model testing the relationship between the number of identified dinoRNAV EVE-containing scaffolds, dinoflagellate genus, and genome quality metrics. Included predictor variables: dinoflagellate genus ("Genus"), N50 score ("N50"), number of bases in the genome ("N_bases"), and number of complete BUSCOs ("N_complete BUSCOs"). Significant predictor variables were identified using an F-test with the function Anova. Model assumptions were visually checked.

File title: Supplemental Data 9
Description: Results from pairwise comparisons of dinoRNAV EVE detections between Symbiodiniaceae genera indicating a significant difference between *Symbiodinium* and *Cladocopium.* *P value adjustment: tukey method for comparing a family of 6 estimates

File title: Supplemental Data 10
Description: Identity and distance of nearest annotated predicted ORFs to identified dinoRNAV EVEs on *Symbiodiniaceae microadriaticum* chromosomes. Positive values indicate upstream, while negative values indicate downstream genes of dinoRNAV EVE integrations (in bp). Likewise, +1 and +2 indicate first and second predicted gene upstream of integration, while -1 and -2 indicate first and second downstream, respectively. *indicates a relict dinoSL within 500 nucleotides of EVE.

File title: Supplemental Data 11
Description: Annotation of genes proximal to putative dinoRNAV EVEs on genome scaffolds. Genome scaffolds annotated with maker3 with repeatmasking and augustus gene prediction using the zebrafish gene model. Protein annotations were inferred with blastp against a hybrid database containing the protein version of the Reference Viral database and the uniprot_spot database (blastp -query $x -db $db -evalue 1e-7 -max_hsps 1 -max_target_seqs 1 -outfmt 6 -out "$name"_output.blastp -num_threads 23) and protein families were inferred with Interproscan w/ HMMER and the PFAM database (interproscan.sh -appl pfam -dp -f TSV -goterms -iprlookup -pa -t p -i $x -o "$name"_output.iprscan -cpu 23).

File Title: Supplemental Data 12
Description: Relict dinoflagellate spliced leaders (dinoSL) within 500bp up- and downstream dinoRNAV *RdRp* EVEs on *Symbiodinium microadriaticum* chromosomes.

File title: Supplemental Data 13
Description: Identification of dinoRNAV-like sequences in publicly accessible transcriptomes of Symbiodiniaceae. Table includes the dinoflagellate species, accession numbers, site link, and number of potential dinoRNAV-like sequences. Transcriptomes were annotated utilizing BLASTx against dinornavirus genomes (Supplemental Table 1B), blastn against the nonredundant database, and manually curated. High confidence sequences are those that contain sobeliviral sequences after ORF calling and annotation (as above), or no matches to the nr database. Potential sequences are those that match reference sequences (Supplemental Data 14), but not the nr database, but may be more fragmented than high confidence sequences.

File title: Supplemental Data 14
Description: Reference database of accession numbers (including HcRNAV, +ssRNAV genomes referenced in Levin et al, 2017, and other dinornavirus-like sequences) utilized to query metagenomes and genomes for endogenous dinoRNAV. Includes both nucleotide (nt) and amino-acid coding sequences

(CDS/aa) accession numbers. ‘Source’ indicates if sequence was derived from an environmental source other than a dinoflagellate (e.g. *genomic* – from a viral genome, *envm* - from a source such as sediment, as submitted by Nakayama & Hamaguchi et al, submitted to NCBI 2020).
