# Supplementary material for: Endogenous viral elements reveal associations between a non-retroviral RNA virus and symbiotic dinoflagellate genomes
Source: Commun Biol. 2023 Jun 1;6:566. doi: 10.1038/s42003-023-04917-9 (PMC10235124; doi:10.1038/s42003-023-04917-9)
Supplement: Supplementary file 4 — Reporting Summary [file 42003_2023_4917_MOESM4_ESM.pdf]

nature portfolio

Corresponding author(s): DBPR COMMSBIO-22-1311-T

Last updated by author(s): Feb 2023

Reporting Summary

Nature Portfolio wishes to improve the reproducibility of the work that we publish. This form provides structure for consistency and transparency in reporting. For further information on Nature Portfolio policies, see our Editorial Policies and the Editorial Policy Checklist.

Statistics

For all statistical analyses, confirm that the following items are present in the figure legend, table legend, main text, or Methods section.

n/a	Confirmed	
☐	☒	The exact sample size (*n*) for each experimental group/condition, given as a discrete number and unit of measurement
☐	☒	A statement on whether measurements were taken from distinct samples or whether the same sample was measured repeatedly
☐	☒	The statistical test(s) used AND whether they are one- or two-sided<br>*Only common tests should be described solely by name; describe more complex techniques in the Methods section.*
☒	☐	A description of all covariates tested
☐	☒	A description of any assumptions or corrections, such as tests of normality and adjustment for multiple comparisons
☐	☒	A full description of the statistical parameters including central tendency (e.g. means) or other basic estimates (e.g. regression coefficient) AND variation (e.g. standard deviation) or associated estimates of uncertainty (e.g. confidence intervals)
☐	☒	For null hypothesis testing, the test statistic (e.g. *F*, *t*, *r*) with confidence intervals, effect sizes, degrees of freedom and *P* value noted<br>*Give P values as exact values whenever suitable.*
☒	☐	For Bayesian analysis, information on the choice of priors and Markov chain Monte Carlo settings
☒	☐	For hierarchical and complex designs, identification of the appropriate level for tests and full reporting of outcomes
☒	☐	Estimates of effect sizes (e.g. Cohen's *d*, Pearson's *r*), indicating how they were calculated

Our web collection on statistics for biologists contains articles on many of the points above.

Software and code

Policy information about availability of computer code

Data collection	acquisition of publicly accessible data (accession numbers) and databases detailed in Supplementary Data 1b and 2
Data analysis	Available in Methods and Supplementary Figure 7, including versions

For manuscripts utilizing custom algorithms or software that are central to the research but not yet described in published literature, software must be made available to editors and reviewers. We strongly encourage code deposition in a community repository (e.g. GitHub). See the Nature Portfolio guidelines for submitting code & software for further information.

Data

Policy information about availability of data

All manuscripts must include a data availability statement. This statement should provide the following information, where applicable:

- Accession codes, unique identifiers, or web links for publicly available datasets
- A description of any restrictions on data availability
- For clinical datasets or third party data, please ensure that the statement adheres to our policy

Original data is available as a component of a larger dataset here: https://zenodo.org/record/6299409#.YkRwrTdBzjA and unique identifiers can be found in Supplementary Data 1a and https://doi.org/10.5281/zenodo.7839794. Publicly accessible data (including accession numbers) can be found with unique identifiers in Supplementary Data 1b.

Field-specific reporting

Please select the one below that is the best fit for your research. If you are not sure, read the appropriate sections before making your selection.

☐ Life sciences ☐ Behavioural & social sciences ☒ Ecological, evolutionary & environmental sciences

For a reference copy of the document with all sections, see nature.com/documents/nr-reporting-summary-flat.pdf

Ecological, evolutionary & environmental sciences study design

All studies must disclose on these points even when the disclosure is negative.

Study description	Full description of this study and data utilized is available in the methods and supplemental material. Briefly, 269 newly sequenced cnidarian metagenomes with affiliated ITS2 amplicon libraries from three species of corals across islands in the Pacific ocean were leveraged to identify EVEs. Metadata associated with these libraries can be found here: https://zenodo.org/record/6299409#.YkRwrTdBzjA with a full protocol of all methodology submitted to Scientific Data. These libraries were paired with additional publicly accessible metagenomes (n= 150) and assembled genomes (of n=25 coral and n=19 dinoflagellate), with accession numbers accessible in Supplementary Data 1 and https://doi.org/10.5281/zenodo.7839794.
Research sample	provided in the following file https://zenodo.org/record/6299409#.YkRwrTdBzjA (paired with rationale in Methods)
Sampling strategy	Bioinformatic sampling and processing available in Supplementary FIgure 1, including sample size calculations. Examination of sequencing depth (to rule out library bias in hydrocoral libraries) available in Supplementary Table 3, precluding methodological bias towards dinoRNAV prevalence among these libraries. Genome completeness scores are available in Supplementary table 5 and depict that there is no statistical relationship between genome completeness and EVE detection. We examined all metagenomes with the identified EVE and all available scaffold- and chromosome-level Symbiodinaceae genomes at the time of analysis.
Data collection	Provided in the following file https://zenodo.org/record/6299409#.YkRwrTdBzjA with unique identifiers in Supplemental Table 1a or provided in Supplemental Table 1b, 2.
Timing and spatial scale	Provided in the following file https://zenodo.org/record/6299409#.YkRwrTdBzjA with unique identifiers in Supplemental Table 1a or provided in Supplemental Table 1b, 2.<br><br>Full description of original field collection will be provided with manuscript presenting the complete protocol and methodology of all Tara pacific sampling, submitted to Scientific Data
Data exclusions	*If no data were excluded from the analyses, state so OR if data were excluded, describe the exclusions and the rationale behind them, indicating whether exclusion criteria were pre-established.*
Reproducibility	*Describe the measures taken to verify the reproducibility of experimental findings. For each experiment, note whether any attempts to repeat the experiment failed OR state that all attempts to repeat the experiment were successful.*
Randomization	*Describe how samples/organisms/participants were allocated into groups. If allocation was not random, describe how covariates were controlled. If this is not relevant to your study, explain why.*
Blinding	*Describe the extent of blinding used during data acquisition and analysis. If blinding was not possible, describe why OR explain why blinding was not relevant to your study.*

Did the study involve field work? ☒ Yes ☐ No

Field work, collection and transport

Field conditions	Field condition metadata of original samples provided here: https://zenodo.org/record/6299409#.YkRwrTdBzjA
Location	Location metadata of original samples provided here, including geographical points : https://zenodo.org/record/6299409#.YkRwrTdBzjA
Access & import/export	Sampling permit for GUAM under the reference 'U2021-023' delivered by the Marine Scientific Research Coordinator Office of Ocean and Polar Affairs – United States Department of State Bureau of Oceans and International Environmental and Scientific Affairs on the 27/10/2021; Sampling permit for AMERICAN SAMOA under the reference 'U2021-022' delivered by the Marine Scientific Research CoordinatorOffice of Ocean and Polar Affairs – United States Department of StateBureau of Oceans and InternationalEnvironmental and Scientific Affairs on the 27/10/2021; Sampling permit for NIUE under the reference '34/16' delivered by the Government of Niue – Office for External Affairs on the 17/11/2016; Sampling permit for COOK under the reference '11-16' delivered by the Foundation for National Research – Cook Island Research Committee – Office of the Prime Minister – Elizabeth Wright-Koteka (Chairperson) on the 12/09/2016; Sampling permit for CLIPPERTON under the reference 'HC/1195/CAB' delivered by the Haut-Commissariat de la République Polynésie Française on the 13/06/2018; Sampling permit for NEW-CALEDONIA (CHESTERFIELD) under the reference 'Arrêté n°2017-2069/GNC' delivered by the Haut-Commissariat de la République en Nouvelle-Calédonie – Gouvernement de Nouvelle-Calédonie – République Française on the 29/08/2017; Sampling permit for UNITED-KINGDOM (PITCAIRN ISLANDS) under the reference 'N/A' delivered by the Government of Pitcairn islands /"Environmental, Conservation & Natural Resources Division Manager" // Christian Michele on the 25/02/2016; Sampling permit for CHILE under the reference '13270/24/457/Vrs' delivered by

	the Servicio Hidrografico y Oceanografico de la Armada de Chile (SHOA) – Patricio Carrasco Hellwig Contraalmirante Director on the 29/08/2016; Sampling permit for COLOMBIA under the reference 'N°009' delivered by the MINISTERIO DE AMBIENTE Y DESARROLLO SOSTENIBLEPARQUES NACIONALES NATURALES DE COLOMBIA on the 04/03/2016; Sampling permit for PANAMA under the reference 'SE/AP-18-16' delivered by the Direccion de Areas Protegidas y Vida Silvestre - LIC. Samuel Valdez Diaz Director - Ministerio de Ambiente – Republica de Panama on the 13/06/2016; Sampling permit for PANAMA under the reference '2016-0701-2019-2' delivered by the Smithsonian Tropical Research Institute Instituto Smithsonian de Investigaciones Tropicales - STRI Animal Care and Use Committee (ACUC) on the 28/06/2016; Sampling permit for PANAMA under the reference '2016-0701-2019-2-A1' delivered by the Smithsonian Tropical Research Institute Instituto Smithsonian de Investigaciones Tropicales - STRI Animal Care and Use Committee (ACUC) on the 21/06/2018
Disturbance	sampling was restricted to the minimal amount of material and did not involved environmentally destructive methods

Reporting for specific materials, systems and methods

We require information from authors about some types of materials, experimental systems and methods used in many studies. Here, indicate whether each material, system or method listed is relevant to your study. If you are not sure if a list item applies to your research, read the appropriate section before selecting a response.

Materials & experimental systems

n/a	Involved in the study
☒	☐ Antibodies
☒	☐ Eukaryotic cell lines
☒	☐ Palaeontology and archaeology
☐	☒ Animals and other organisms
☒	☐ Human research participants
☒	☐ Clinical data
☒	☐ Dual use research of concern

Methods

n/a	Involved in the study
☒	☐ ChIP-seq
☒	☐ Flow cytometry
☒	☐ MRI-based neuroimaging

Animals and other organisms

Policy information about studies involving animals; ARRIVE guidelines recommended for reporting animal research

Laboratory animals	N/A
Wild animals	organisms sampled were invertebrates and only vegetative/clonal fragments were collected
Field-collected samples	Samples were preserved immediately for sequencing. see: https://zenodo.org/record/6299409#.YkRwrTdBzjA
Ethics oversight	N/A (see permits)

Note that full information on the approval of the study protocol must also be provided in the manuscript.
